# An Augmented High-Dimensional Graphical Lasso Method to Incorporate Prior Biological Knowledge for Global Network Learning

**DOI:** 10.3389/fgene.2021.760299

**Published:** 2022-01-27

**Authors:** Yonghua Zhuang, Fuyong Xing, Debashis Ghosh, Farnoush Banaei-Kashani, Russell P. Bowler, Katerina Kechris

**Affiliations:** ^1^ Department of Biostatistics and Informatics, University of Colorado Anschutz Medical Campus, Aurora, CO, United States; ^2^ Department of Computer Science and Engineering, University of Colorado Denver, Denver, CO, United States; ^3^ National Jewish Health, Denver, CO, United States

**Keywords:** graphical Lasso, Gaussian graphical model, protein-protein interaction, gene network, systems biology

## Abstract

Biological networks are often inferred through Gaussian graphical models (GGMs) using gene or protein expression data only. GGMs identify conditional dependence by estimating a precision matrix between genes or proteins. However, conventional GGM approaches often ignore prior knowledge about protein-protein interactions (PPI). Recently, several groups have extended GGM to weighted graphical Lasso (wGlasso) and network-based gene set analysis (Netgsa) and have demonstrated the advantages of incorporating PPI information. However, these methods are either computationally intractable for large-scale data, or disregard weights in the PPI networks. To address these shortcomings, we extended the Netgsa approach and developed an augmented high-dimensional graphical Lasso (AhGlasso) method to incorporate edge weights in known PPI with omics data for global network learning. This new method outperforms weighted graphical Lasso-based algorithms with respect to computational time in simulated large-scale data settings while achieving better or comparable prediction accuracy of node connections. The total runtime of AhGlasso is approximately five times faster than weighted Glasso methods when the graph size ranges from 1,000 to 3,000 with a fixed sample size (*n* = 300). The runtime difference between AhGlasso and weighted Glasso increases when the graph size increases. Using proteomic data from a study on chronic obstructive pulmonary disease, we demonstrate that AhGlasso improves protein network inference compared to the Netgsa approach by incorporating PPI information.

## 1 Introduction

Networks are a useful framework for representing relationships in biological and disease pathways (Zhang et al., 2014). Understanding complex biological networks including protein-protein interaction (PPI) networks is a fundamental and challenging issue in computational and systems biology (Kuchaiev et al., 2009). Known protein-protein interactions have been collected from numerous sources, including experimental data, computational prediction methods, and public text collections to form a global network. STRING (Search Tool for the Retrieval of Interacting Genes/Proteins) is one of the most comprehensive protein association databases and it includes direct (physical) and indirect (functional) associations (Szklarczyk et al., 2016). Although STRING is continually being updated, the protein-protein interactions are still not complete and accurate due to potential errors and missing information in current high-throughput assays (Huttlin et al., 2017). In addition, protein interactions and pathways associated with specific diseases in STRING may be limited. To build a more complete and specific protein-protein interaction network related to diseases of interest, we need to reconstruct networks based on study-specific co-expression data, in addition to prior knowledge of protein-protein interactions.

Gene networks are commonly inferred using co-expression data (Saelens et al., 2018). One popular expression-based network reconstruction method is weighted gene co-expression network analysis (WGCNA) (Langfelder and Horvath, 2008). It was originally designed for microarray expression measurements. More recently, it has been extended for sequencing expression data, as well as other data, such as proteomic and metabolomic (DiLeo et al., 2011; Shirasaki et al., 2012; Zhang et al., 2013; Langfelder et al., 2016). While WGCNA has gained popularity, it was originally designed for a single data type but more recently has been extended for integrating multiple data types (Mamdani et al., 2015), by first constructing relevant homogeneous networks in parallel and then combining the separate networks. However, it is not clear how to best combine networks based on different data types and how to incorporate known pathways or protein/genetic interaction information.

Another popular expression-based network reconstruction approach is modeling gene interactions using a Gaussian graphical model (GGM) (Dobra et al., 2004). Under the assumption of multivariate normality of gene expression data, the GGM uses the inverse of the gene covariance matrix as a measure for gene associations. For many genes, the associations are usually very sparse. One popular method to estimate a sparse network is the graphical Lasso algorithm (Mamdani et al., 2015). Similar to WGCNA, GGMs are widely used in biological applications for network graph construction but often ignore the known protein/genetic interaction. Recently, this approach was extended to incorporate partially known information with a weighted graphical Lasso (Li and Jackson, 2015; Zuo et al., 2017). It has been demonstrated that weighted Glasso significantly improved the prediction accuracy of protein-protein interactions. However, the graphical Lasso can be computationally expensive for a large-scale feature space and therefore limited for global network learning using high-dimensional omics data (Fattahi and Sojoudi, 2019).

In addition to the weighted graphical Lasso, Jing Ma *et al.* developed a network-based gene set analysis (Netgsa) approach for network-based enrichment analysis by incorporating prior pathway information (Ma et al., 2016). The Netgsa approach combines the neighborhood selection technique (Meinshausen and Buhlmann, 2006) with constrained maximum likelihood estimation using the graphical Lasso algorithm (Friedman et al., 2008). It exploits the fact that the estimated neighbors of each node using neighborhood selection coincide with the nonzero entries of the inverse covariance matrix, resulting in high accuracy and fast computation. The Netgsa method was designed to take prior binary node interaction information in one or a few pathways and estimate the edge strengths based on the current data. However, the Netgsa approach does not account for edge weights (e.g., interaction strengths) of known but incomplete protein-protein interactions. In addition, whether the hybrid approach of combining neighborhood selection and maximum likelihood estimation outperforms conventional weighted Glasso has not been well studied.

Besides GGM-based network analysis, there are some recent advances for incorporating prior knowledge in protein or gene network reconstruction. These newly developed methods include Multi-Level PPINs reconstruction (MLPR) (Xu et al., 2018), Diffuse2Direct approach to orient a network (Silverbush and Sharan, 2019), Ensemble Deep Neural Networks with Attention Mechanism (EnAmDNN) (Li et al., 2020), and prior network-dependent gene network inference (pGNI) (Wang et al., 2021). The MLPR method was designed for protein complexes detection through a random walk on the fingerprint similarity networks (Xu et al., 2018). Diffuse2Direct is a diffusion-based method to incorporate prior knowledge and orient an undirected or a partially directed network (Silverbush and Sharan, 2019). The EnAmDNN approach is a deep ensemble learning method to combine multiple models for network construction (Li et al., 2020). The pGNI method was designed to incorporate the modular structures for protein-protein network reconstruction (Xu et al., 2018). However, these newly developed methods are not based on conditional correlation and precision matrix for graph construction. As standard correlation networks, these methods do not take into account the conditional dependencies of proteins, which could lead to potential bias.

In our proposed method, Augmented High-Dimensional Graphical Lasso model (AhGlasso), we first extend the Netgsa hybrid approach to incorporate the edge weights of prior known protein-protein relationships and omics data for global network learning. Then, we implement a screening based on the standard Pearson correlation to further speed up computation. We compare our proposed method with Netgsa and weighted Glasso-based methods in terms of computation time and accuracy with simulated data. Of note, we do not compare AhGlasso with MLPR, Diffuse2Direct, EnAmDNN and pGNIC since these four methods do not provide conditional correlation outputs. Finally, we illustrate an application of AhGlasso on protein expression data from the COPDGene study.

## 2 Materials and Methods

### 2.1 Graph Structure Learning With Augmented Graphical Lasso

There are several methods for network structure learning including correlation networks and Gaussian graphical models (GGM). The correlation network method is based on the covariance matrix Σ. Σ_*i*,*j*_ = 0 means that *X*_*i*_ and *X*_*j*_ are marginally independent without observing other variables. However, this kind of independence is hard to find in real-world problems. Instead, GGM is more appropriate since it is based on conditional correlations and the precision matrix. Compared with the more standard correlation network, the conditional independence correlation coefficient is a more sophisticated dependence measure and may be more suitable for modeling real-world biological networks.

To obtain the precision matrix, a common assumption is that the precision matrix Q is sparse. For example, genes are only assumed to interact with a small subset of other genes. Based on the above assumption, we could apply a neighbor selection approach developed by Meinshausen and Bühlmann to learn the relationship between two nodes with Lasso regression, which allows zero parameters through a penalty (Meinshausen and Buhlmann, 2006). Here we take node *i* as an example to illustrate how to identify one node’s neighbors.

$${\hat{\beta }}_{node_{i}}=\underset{\beta_{node_{i}}}{\arg\min}\|Y-X\beta_{node_{i}}{\|}_{2}^{2}+\lambda \|\beta_{node_{i}}{\|}_{1},$$ (1)

where **Y** is the expression value of *node*_*i*_, **X** denotes the expression values of all the other nodes, ${\hat{\beta }}_{node_{i}}$ is a vector of estimated coefficients from the Lasso regression, and *λ* is the sparsity parameter for the Lasso regression. The Lasso regression is repeated for each node and determines whether pairwise nodes are conditionally independent or not (Meinshausen and Buhlmann, 2006). Although the neighborhood selection method is remarkably fast, it is an approximation method for estimating sparse networks. Friedman et al., developed the Graphical Lasso to address sparse inverse covariance estimation with constrained maximum likelihood estimation (Friedman et al., 2008).

GGMs including neighborhood-selection algorithm and Graphical Lasso are widely used in biological applications for network graph construction but ignores known protein/genetic interactions. This approach was recently extended to incorporate partially known information with a weighted graphical Lasso (Li and Jackson, 2015; Zuo et al., 2017). However, it has been known that the Graphical Lasso can be computationally intractable for high-dimensional omics data (Zhang et al., 2018; Fattahi and Sojoudi, 2019). In addition, a Network-Based Gene Set analysis (Netgsa) approach was developed to incorporate prior pathway information (Ma et al., 2016). The Netgsa approach combines the neighborhood selection technique (Meinshausen and Buhlmann, 2006) with constrained maximum likelihood estimation (Friedman et al., 2008). The Netgsa approach was designed to incorporate known binary interaction information in one or a few pathways and estimate the edge strengths. However, it does not take edge weights (e.g., interaction strength) into account.

**Algorithm 1:** Graph structure learning with augmented high-dimensional graphical Lasso.

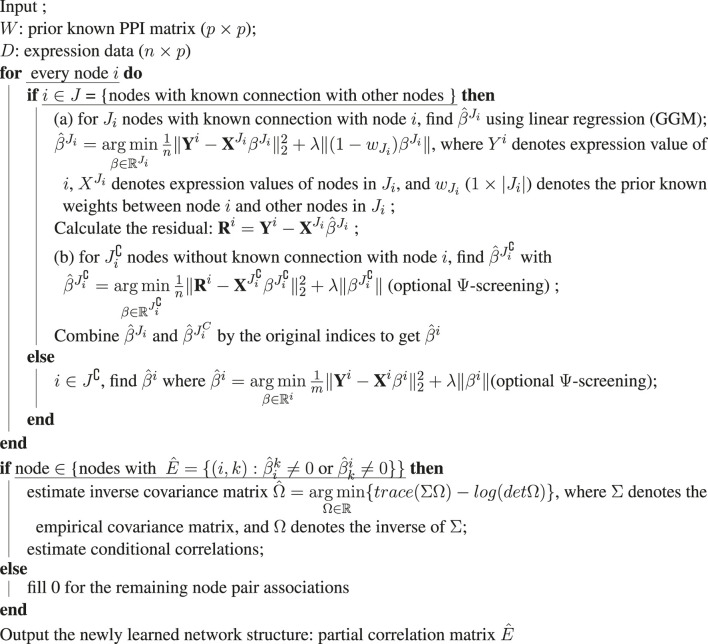

**Algorithm 2:** *λ* optimization in augmented high-dimensional graphical Lasso.

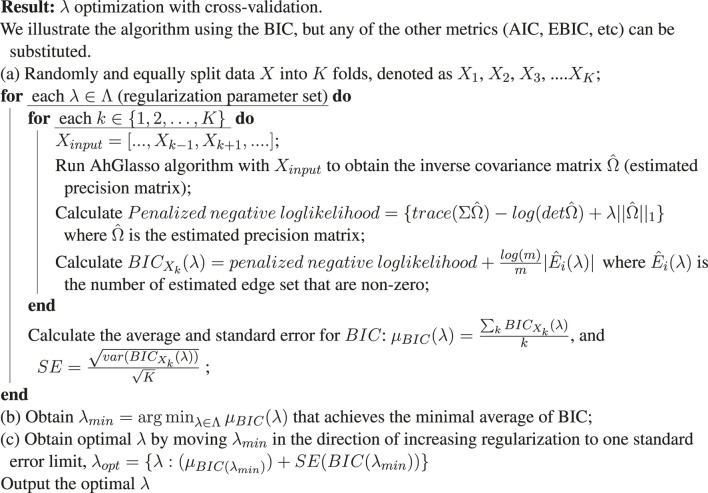

In our proposed method, AhGlasso, we first extend the Netgsa approach and incorporate the edge weights of prior known but incomplete protein-protein relationships from STRING as shown in Algorithm 1 to reconstruct the global network. The method combines the neighborhood selection strategy with constrained maximum likelihood estimation using Graphical Lasso algorithm to efficiently reconstruct the global network. We also apply Ψ-screening as discussed below to speed up computation. The input for AhGlasso is expression data ***D***_*n*×*p*_, where *n* denotes the number of samples and *p* denotes the number of nodes (genes or proteins). In addition, the input is a prior known PPI matrix ***W***_*p*×*p*_ = [*w*_*ij*_], where *i*, *j* = 1, 2, … , *p* and *w*_*ij*_ denotes the edge weights, and for diagonal entries *w*_*ii*_ = 0. ***P*** is the set of all nodes in the network graph. ***J*** denotes the set of nodes which have at least one connection with other nodes in the prior known PPI matrix and ***J*** ^∁^ denotes the set of isolated nodes (***J*** ^∁^ = ***P*** ∖ ***J***). For node *i* in ***J***, ***J***_*i*_ indicates other nodes which have prior known connections with node *i*, while $J_{i}^{∁}$ denotes other nodes that are not connected with node *i*. Of note, both ***J***_*i*_ and $J_{i}^{∁}$ do not include the *i*th node. In the expression data (***D*** matrix), ***Y*** ^*i*^ is a column vector (*n* × 1) of expression data for node *i* (i.e., ***D***_.,*i*_) and $X^{J_{i}}$ denotes the expression data for the nodes in ***J***_*i*_, which are connected to node *i*. The output of the algorithm is the conditional correlation matrix between nodes, ${\hat{E}}_{p\times p}$ .

### 2.2 Network Structure Learning With Ψ-screening and Ψ Partial Correlation Coefficient

Liang *et al.* proposed an equivalent measure of partial correlation coefficients for GGM under the assumption of the Markov property and adjacency faithfulness (Liang et al., 2015). They defined the set of nodes *v* where the edge weight to node *i* is greater than *γ* as ${\hat{E}}_{i}\left(\gamma \right)=\left\{v:|{\hat{e}}_{iv}|>\gamma \right\}$ , where $\hat{e}$ is the pair partial correlation of nodes *i* and *v*, and *γ* denotes a threshold value. Additional sets are defined as ${\hat{E}}_{i,-k}\left(\gamma \right)=\left\{v:|{\hat{e}}_{iv}|>\gamma \right\}\backslash \left\{k\right\}$ , and ${\hat{E}}_{k,-i}\left(\gamma \right)=\left\{v:|{\hat{e}}_{kv}|>\gamma \right\}\backslash \left\{i\right\}$ , where *k* denotes a node in network graph not equal to *i*. The partial correlation coefficient Ψ_*ik*_ was defined by Ψ_*ik*_ = [*ψ*_*ik*_], where $\psi_{ik}={\hat{E}}_{i,-k}$ if $\left|{\hat{E}}_{i,-k}|<|{\hat{E}}_{k,-i}\right|$ and $\psi_{ik}={\hat{E}}_{k,-i}$ otherwise. With the partial correlation coefficients, the network structure could be learned with the Ψ algorithm (Supplement) including correlation screening (Liang et al., 2015). For correlation screening, we could reduce the size of the neighborhood by removing the nodes having a lower correlation (in absolute value). Similar to the *huge* R library (Zhao et al., 2012), we adapted the correlation screening step in the Ψ-algorithm to AhGlasso to reduce the size of potential neighborhood in Algorithm 1 and speed up the network estimation. For example, for *i* ∈ *J* ^∁^, we find ${\hat{\beta }}^{i}=\underset{\beta \in R^{i}}{\mathrm{arg min}}\frac{1}{m}\|Y^{i}-X^{i}\beta^{i}{\|}_{2}^{2}+\lambda \|\beta^{i}\|$ . Instead of computing all potential neighbor nodes ${\hat{\beta }}^{i}$ with Lasso, we could reduce the size of the *i* neighborhood by removing the nodes having a low Pearson correlation (in absolute value). In other words, we only need to find the potential neighbor nodes with high Pearson correlation (absolute value > *λ*) with node *i*.

### 2.3 Hyper-Parameter Tuning and Model Selection

Like other Lasso-based optimization procedures, the sparsity parameter *λ* is crucial for AhGlasso because it controls the sparsity of network prediction. If the lambda value is greater than the optimum, we could get an over-sparse network estimation. If the lambda value is smaller than the optimum, we could get over-dense network estimation. There is no consensus on how to select the *λ* hyper-parameter, which is an active research area. The *λ* parameter could be tuned by log-likelihood, Akaike Information Criteria (AIC), Bayesian Information Criteria (BIC), or the Extended Bayesian Information Criteria (EBIC), and with or without cross-validation. AIC and EBIC often lead to an over-sparse network, while the log-likelihood may result in a too dense network since there is no penalty for the number of edges. Although BIC works well in general low-dimensional scale-free networks, it could lead to under-fitting and an over-sparse network, especially when the network graph is large or does not have the scale-free property. Although real-world networks are often claimed to be scale-free, strongly scale-free structure is rare even in biological domains (Broido and Clauset, 2019). Selecting the criteria for model selection is difficult and depends heavily on uncheckable or difficult-to-check assumptions on the data generating process. K fold cross-validation (CV) provides a potential tool to solve this challenge. In this study, we evaluated different cross-validation options (AIC, BIC, EBIC) and the standard BIC for parameter optimization in a scale-free and non-scale-free network. The model fitting error is AIC, BIC or EBIC for the *λ* parameter selection. The one standard error rule was also adapted to compare models with different numbers of parameters to select the most parsimonious model with low error (Hastie et al., 2019). Specifically, the simplest model whose mean error falls within one standard deviation from the smallest average (e.g., minimal mean of BIC in CV) achieved for the respective metric (e.g., BIC) was chosen (Algorithm 2). Of note, the AIC, BIC, and EBIC are maximum likelihood estimate driven and penalize free parameters in an effort to combat overfitting. In Graphical Lasso model selection, AIC, BIC, and EBIC are often calculated based on the log Likelihood but with different penalization strategies for the number of parameters (Li and Jackson, 2015; Ma et al., 2016; Zuo et al., 2017). Since the Glasso estimates the inverse matrix based on penalized log Likelihood, we calculate AIC, BIC, and EBIC based on the penalized log Likelihood instead.

In order to find the optimal regularization parameter, *λ*, networks were estimated under a sequence of *λ* values. The upper bound (*λ*_max_, representing maximum value of *λ*) of the regularization parameter which makes all estimates equal to 0 was calculated with the *huge* package (Zhao et al., 2012). With predefined *λ* minimal ratio (such as 0.01), we can calculate *λ*_min_ (representing minimal value of *λ*), which is equal to the *λ* minimal ratio × *λ*_max_. A sequence of *λ* candidates with a predefined number (such as 40) were generated starting from *λ*_min_ to *λ*_max_ in a log scale for a grid search.

### 2.4 Data Simulation

**FIGURE 1:**
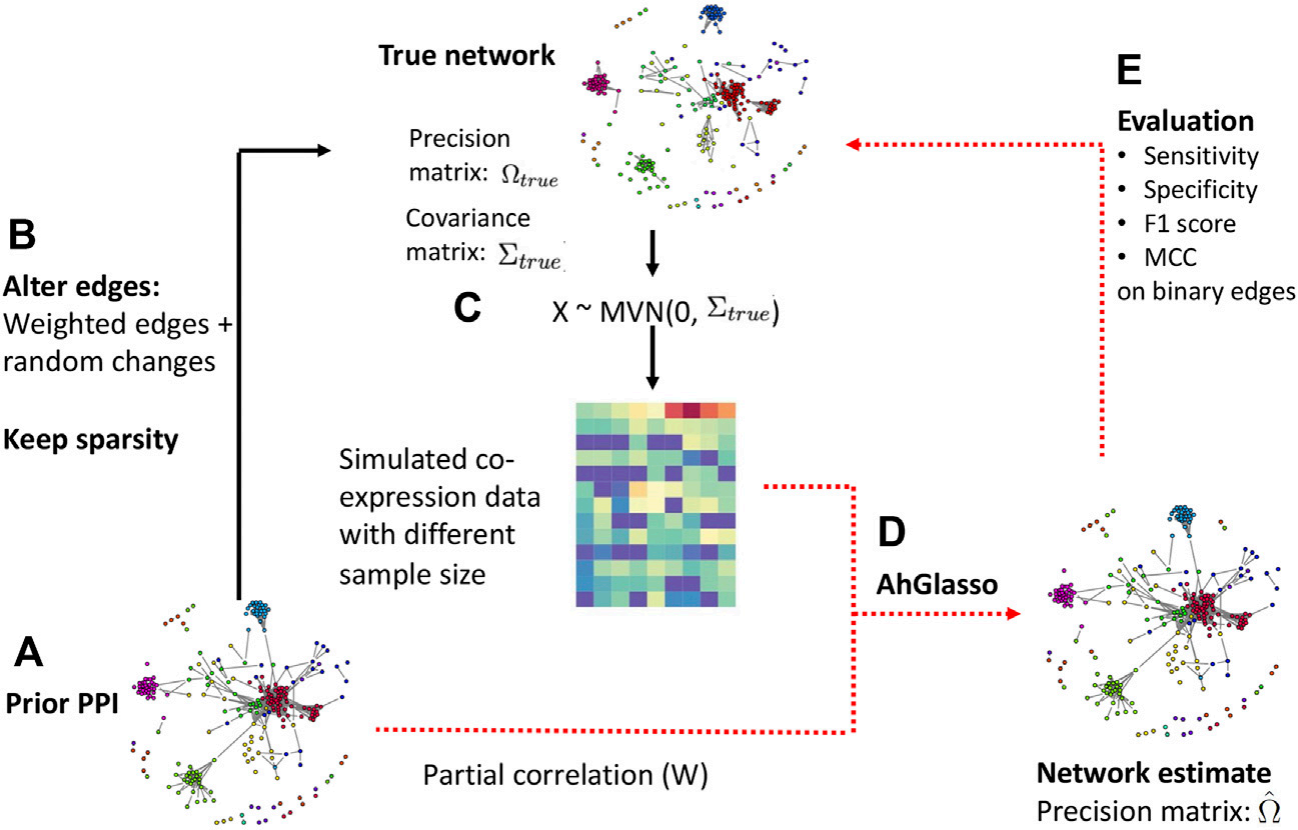
Flowchart of data simulation and method evaluation. Step **(A)**: a scale-free prior network was built by inputting the node number p (graph size). With the prior scale-free network and its precision matrix Ω, the partial correlation can be calculated. The absolute value of partial correlation serves as prior knowledge (prior PPI) for upcoming simulations. Step **(B)**: we altered network connections by adding random changes but the sparsity was kept at a similar level unless otherwise specified. Strong node connections were likely kept due to their higher absolute value in the precision matrix and were less likely to become 0. Step **(C)**: with altered precision matrix (Ω_*true*_) and its corresponding covariance matrix (Σ_*true*_), we simulated expression data *X*_*n*×*p*_ ∼ *N* (0, Σ_*true*_). Of note, we ensured the covariance matrix to be positive definite based on the Frobenius norm. Step **(D)**: incorporating prior PPI and simulated dataset, we estimated the target network (i.e., adjacency matrix) with our proposed method. Step **(E)**: the prediction performance was evaluated with sensitivity, F1 score, and MCC.

Scale-free biological networks often have two properties: 1) the node degree follows a power-law distribution and 2) the interactions of proteins/genes are sparse. Therefore, we simulated sparse networks with the scale-free property using the R packages *huge* (Zhao et al., 2012) and *fastnet* (Dong et al., 2016) to mimic biological networks (Figure 1). The simulation procedures are detailed in the Figure 1 legend. The density of the simulated networks ranged from 2 to 4*%*, which was similar to observed densities of protein-protein interactions in the STRING (Search Tool for the Retrieval of Interacting Genes/Proteins) database for many organisms (Szklarczyk et al., 2016; Ashtiani et al., 2018). For example, when the node number is 1,000, the number of connected edges in 2*%* density of the simulated network $=\frac{1000\times \left(1000-1\right)}{2}\times 0.02$ of connections exist. Of note, the simulated scale-free graph with *huge* R package tends to be very sparse (less than 0.005) when the graph size is larger than 500. We simulated the scale-free graph structure with the modified “net.barabasi.albert” function in *fastnet* R package (Dong et al., 2016). Once the scale-free network is built, *huge* creates the true precision matrix Ω and the partial correlation can be calculated based on the precision matrix. The absolute value of the partial correlation serves as the prior knowledge (prior PPI) for upcoming simulations. We use the absolute value since the direction of many protein-protein interactions in STRING is not specified.

We altered network connections by adding random changes on the precision matrix but keeping the sparsity at a similar level because the biological network is dynamic. For simplicity, we assume the total number of newly appearing interactions is close to the total number of existing interactions that get lost. The precision matrix controls the magnitude of partial correlations. The original off-diagonal elements of the precision matrix range from 0.2 to 1. The symmetric uniform distributed random values range from −0.5 to 0.5. Strong node connections were likely kept due to their higher values in the precision matrix. If the absolute values of altered elements in the precision matrix were less than 0.2, we reset them to 0. The altered precision matrix and its corresponding covariance matrix served as the target network we would infer and compare to evaluate method performances. We defined them as “true” precision matrix and covariance matrix for simplicity. With the altered precision matrix (Ω_*true*_) and its corresponding covariance matrix (Σ_*true*_), we could simulate expression data *X*_*n*×*p*_ ∼ *N* (0, Σ_*true*_). Of note, we obtain a positive definite precision matrix with the Frobenius norm through the *huge* R package (Zhao et al., 2012). Specifically, the smallest eigenvalue of the precision matrix Ω_*true*_, denoted by *λ*_1_ is computed. Then we set the precision matrix equal to Ω + (|*λ*_1_| + 0.1)*I*. The covariance matrix is then computed to generate multivariate normal data.

We created simulation datasets with various *p* (graph size) and *n* (sample size) as well as different overlapping degree between the prior known network and the target true network. The overlapping degree between the prior known network and the target true network was defined as *ρ*. The *ρ* was calculated by comparing the prior known precision matrix and target precision matrix in binary format (i.e., non-zero weights were converted to one for simplified calculation). The range of *ρ* between 0 and 100 was explored. Specially, adding random changes on the precision matrix above leads to a certain degree of *ρ* change. To allow for a variety of overlapping percentages, we randomly replaced some non-zero edges in precision matrix to become 0 with predefined mutation percentages while the same number of randomly selected zero edges were replaced to be non-zero. In other words, a similar sparsity level of the network was kept. The *ρ* degree was determined with the altered network and the prior network at the end. Using the prior PPI and simulated dataset, we estimated the target network with our proposed new method. We also estimated the network without prior PPI information as the baseline control. We implemented two published weighted graphical Lasso approaches and the Netgsa method for comparison (Li and Jackson, 2015; Ma et al., 2016; Zuo et al., 2017). In the Netgsa implementation, non-zero weights in prior PPI were converted to 1.

We created simulation datasets with various *p* (graph size, from 400 to 3000) and *n* (sample size, from 100 to 1000) for evaluating different *λ* tuning criteria and comparing different methods. In order to systematically evaluate AhGlasso with prior knowledge, simulations were performed with a variety of overlapping percentages between prior knowledge and target true network (ranging from 0 to 100*%*).

In reality, the prior PPI is often partially-known but not totally noisy. We also investigated the method performance when the prior network is a subset of the true network. We randomly removed the connected edges in the prior network under the predefined subset percentage (ranging from 10 to 100*%*) of the target network. If the subset percentage for the prior network is 100*%*, the prior network is the same as the target network. If the subset percentage for prior network is 50*%*, the density of the prior network is 50*%* of the density of the target network.

### 2.5 Model Evaluation

To measure the accuracy of the estimation and compare the method performances, we focused on the accuracy of whether an edge existed in the true graph and was also estimated to be non-zero. That is, we converted non-zero estimated edges to one in the edge matrix and counted whether in the true graph this edge existed (and vice versa) to define negative or positive prediction. We use several metrics including sensitivity, specificity, F1 score, and Matthews correlation coefficient (MCC). The F1 score can be interpreted as a weighted average of the precision and recall and is a suitable measure for imbalanced datasets like sparse networks. The sparse network is one of extremely imbalanced datasets since only a small percent of edges between proteins exist while the majority of elements in the adjacency matrix are 0. F1 score is computed as

$$F1=\frac{2*Precision*Recall}{Precision+Recall}=\frac{2*TP}{2*TP+FP+FN},$$ (2)

where Precision $=\frac{TP}{TP+FP}$ , and Recall = $\frac{TP}{TP+FN}$ . TP, TN, FP, and FN are the number of true positives, true negatives, false positives, and false negatives, respectively.

MCC is another metric for measuring classification performance and is widely used in network prediction. MCC takes into account all four values in the confusion matrix, and a high value (close to 1) means that both classes are predicted well. MCC was calculated as follows based on 2 × 2 contingency table,

$$MCC=\frac{TP\times TN-FP\times FN}{\sqrt{\left(TP+FP\right)\left(FP+FN\right)\left(TN+FP\right)\left(TN+FN\right)}},$$ (3)

where TP, TN, FP, and FN are the number of true positives, true negatives, false positives, and false negatives, respectively.

### 2.6 STRING PPI Database

STRING (http://www.STRING-db.org) is a database of known and predicted protein-protein interactions. It currently covers 5,214,234 proteins from 1133 organisms (Szklarczyk et al., 2016). In STRING, protein-protein pair associations (i.e., the “edge weights” in each network) are represented by confidence scores. The scores indicate the estimated probability that a given interaction is biologically meaningful, specific, and reproducible, given the supporting evidence. There are seven evidence channels in STRING: 1) experiments; 2) database; 3) text-mining; 4) coexpression; 5) neighborhood; 6) fusion; and 7) co-occurrence. The edge scores (weights) between proteins in the STRING PPI database range from 0 to 1, with 1 being the highest possible confidence of interaction. In the COPD study, we retrieved human PPI data from STRING and filtered out the prior interactions with scores less than 0.2.

### 2.7 Proteomics Data in COPDGene Study

As an application of the methodology to real data, we used the proteomics data generated in the COPDGene Study. COPDGene is a multicenter genetic epidemiology study that enrolled 10,198 participants with and without the chronic obstructive pulmonary disease (COPD) between 2007 and 2011 (Phase I study) to identify genetic factors associated with COPD (Ragland et al., 2019). COPD is a disease characterized by reduced lung function and symptoms such as shortness of breath. Five-year follow-up visits took place from 2013 to 2017 (Phase II study). Proteomic profiles were constructed on participants who agreed to participate in the ancillary study of Phase II COPDGene study. All analyses were performed on frozen plasma from p100 tubes. After removing observations that did not pass QC or have no phenotype data, were duplicates, whose primary pulmonary diagnosis was not COPD, were a never smoker, and at Phase 2 reported having a lung transplant or lung volume reduction before Phase 2, there was 1206 subject in Phase 1 and 1010 in Phase 2. The Global Obstructive Lung Disease (GOLD) system was used to grade the severity of airflow limitation: GOLD 0 (controls) and GOLD 1–4 (COPD cases). Our study focuses on the 486 COPD cases (GOLD stage >0) in the Phase 2 study since the inherent protein networks between controls and COPD cases might be different (Mastej et al., 2020).

Proteomic profiling was performed using the SomaScan® platform (Boulder, Colorado) (Candia et al., 2017). The Human Plasma SomaScan® 1.3k kit (SL Part Number 900–00011) was used following the manufacturer’s recommended protocol. Data from all samples passed quality-control criteria and were fit for analysis. To map with the STRING database, the proteomics expression data without one-to-one mapping to gene symbols were removed. For example, if two SomaScan® aptamers map to the same gene symbol, these two aptamers’ corresponding expression data were removed. In addition, some aptamers either detect the expression level of a protein complex or detect the total expression level of several proteins by targeting a shared subunit. These aptamers were removed as well for simplicity. Expression data for 1212 proteins were retained for network construction.

### 2.8 Gene Ontology Enrichment Analysis on Hub Proteins in COPD Associated Network

Using the prior PPI network retrieved from the STRING database, we applied AhGlasso and Netgsa to construct COPD-associated networks. In addition, we also constructed a COPD-associated network without a prior PPI network for comparison. Because there is a lack of ground truth to evaluate the prediction accuracy, we performed Gene Ontology (GO) enrichment analysis with Fisher’s exact test using *the topGO* R package (Alexa and Rahnenführer, 2009) on hub proteins in the COPD-associated networks. Specifically, we analyzed the top 40 hub proteins to identify significantly enriched molecular functions in the updated COPD networks. The hub proteins were defined based on the degree of nodes. Gene Ontology (GO) is a well-known framework for supporting the computational representation of biological systems (Ashburner et al., 2000). It defines a set of concepts used to describe the functions of gene products, and relationships between these concepts. It contains three aspects that hold terms defining the basic concepts of molecular function (MF), biological processes (BP), and cellular components (CC), respectively. Specifically, a GO annotation is an association between a specific gene product and a GO concept. GO was well established and has often been used to evaluate the quality of newly constructed or reconstructed protein-protein interaction networks (Xu et al., 2018; Seyyedsalehi et al., 2021). We focused on BP ontology enrichment analysis since we are interested in what biological processes are involved in COPD. The adjusted *p* values were calculated with Benjamini-Hochberg Procedure for False Positive Rate (FDR) correction. The significant level was set to FDR <0.05.

### 2.9 Statistical Software

Unless otherwise specified, the data manipulation and data analyses were performed using *RStudio* (version 1.2.5019) (RStudio Team, 2019) and R (version 4.0.3) (R Core Team, 2020). The R packages *ggplot2*_1.8.6 (Wickham, 2009), *biomaRt*_2.44.4 (Durinck et al., 2005), *topGO*_2.40.0 (Alexa and Rahnenführer, 2009), *plyr*_1.8.6 (Wickham, 2011), *Netgsa*_3.1.0 (Ma et al., 2016), *Glasso*_1.11 (Friedman et al., 2008) and *huge*_1.3.4.1 (Zhao et al., 2012) were used for data preparation and gene network analysis from differential expression data.

## 3 Results

### 3.1 Model Selection Criteria

**FIGURE 2:**
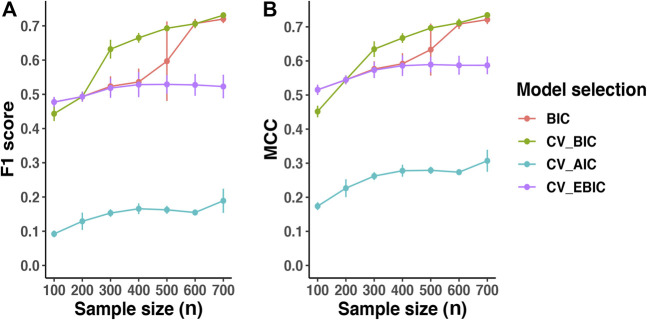
Comparison of Model Selection Criteria. The simulated protein network included 500 (*p*) nodes. The overlapping between prior information and target true network is 50*%*. With the same true network and its corresponding covariance matrix (Σ_*true*_), we created various sizes (*n*) of multiple normal expression data for testing. The *λ* was optimized with regular BIC, cross-validation-based BIC (CV_BIC), cross-validation-based AIC (CV_AIC) and cross-validation-based EBIC (CV_EBIC). The AhGlasso algorithm with the optimal *λ*s derived from different criteria outputs the predicted network. The F1 score and MCC were calculated based on the estimated network and true network. For each simulation setting, the simulations were repeated 5 times. The lines represent the mean scores for the simulated sample size and the error bars represent the standard error of the mean. Of note, similar results were achieved in various *p* and *n* simulations (data not shown).

Since *λ* controls the sparsity of the output network, selecting the *λ* parameter is crucial for Lasso-based approaches like AhGlasso. The *λ* parameter can be selected based on a variety of criteria but there is no consensus on the best criteria. Cross-validation provides a general tool to solve this kind of challenge. For *p* = 500, we found that cross-validation with BIC provides higher accuracy in terms of F1 score (Figure 2A) and MCC (Figure 2B) than BIC without cross-validation when the sample size is between 300 and 600. When the sample size is larger, BIC with or without cross-validation is similar. Cross-validation with BIC outperforms cross-validation with AIC and EBIC. Finally, cross-validation with AIC and EBIC results in an under-fitting model and over-sparse network, leading to a lower F1 score and MCC. Therefore, we chose cross-validation with BIC as model selection criteria to select the *λ* parameter henceforth.

### 3.2 Incorporating Prior Knowledge Significantly Improves Prediction Accuracy

**FIGURE 3:**
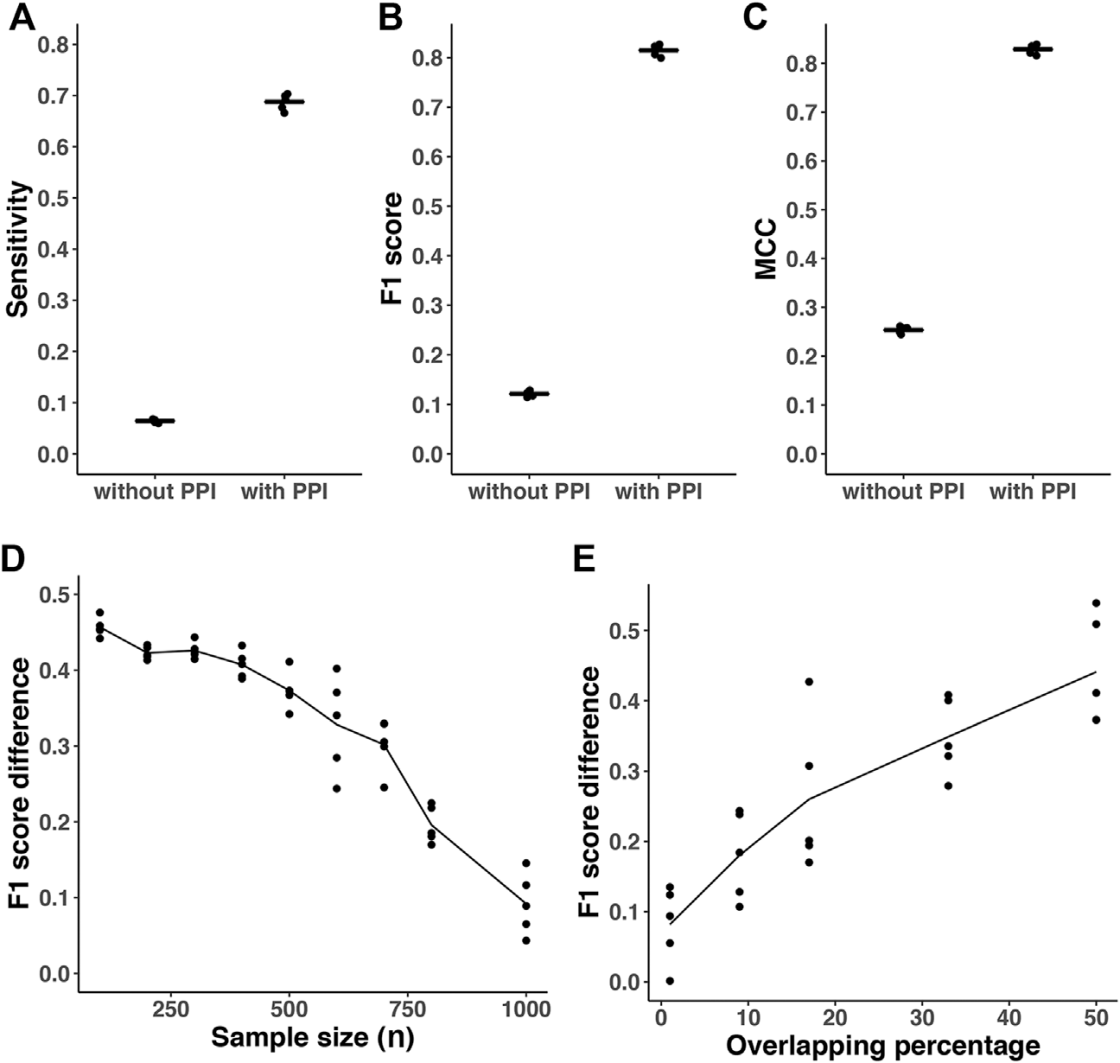
Prediction accuracy comparison with or without incorporating prior information. All results are based on *p* = 500 nodes and *n* = 200. The density of simulated graph is around 2*%*. The *λ* was optimized with cross-validation-based BIC (CV_BIC). The AhGlasso algorithm with the optimal *λ*s output the predicted networks with or without incorporating prior knowledge. **(A–C)** The overlapping degree between prior information and the target true network is 80*%*. The sensitivity **(A)**, F1 score **(B)**, and MCC **(C)** were calculated based on the estimated network and true network. For each simulation setting, the simulations were repeated 5 times. The bars represent the means of corresponding statistics metrics. Pair student’s t-tests were performed to compare the corresponding metrics. All *p* values <0.0001. **(D)**, the overlapping degree between prior information and target true network is 50*%*. A variety of sample sizes of data was simulated as shown in X-axis. The simulation was repeated 5 times for each simulation setting. Y-axis represents the difference of F1 score between the outputs with or without prior knowledge. The lines represent the mean scores and the dots represent the results in each simulation. **(E)**, a variety of overlapping degree between prior information and target true network was simulated as shown in X-axis. Y-axis represents the difference of F1 score between the outputs with or without prior knowledge. The lines represent the mean scores and the dots represent the results in each simulation.

In different simulation settings, we found that the predicted networks based on *a priori* information had significantly greater performance than estimations without *a priori* information (Figure 3). For simulations with *p* = 500, *density* = 2*%*, *overlapping*(*ρ*) = 80*%*, the mean of sensitivity, F1 score and MCC in the estimated networks with prior knowledge are 0.68, 0.81 and 0.82, respectively (Figures 3A–C). However, the corresponding mean of sensitivity, F1 score, and MCC without prior knowledge are only 0.07, 0.12, and 0.25, respectively. The differences of corresponding metrics are statistically significant (*p* < 0.0001, paired student’s t-test). In addition, with fixed 50*%* overlapping, we found that the F1 score difference decreases when the sample size gets larger (Figure 3D). It is expected that when we have a larger sample size, the advantages of incorporating prior information are diminished compared to smaller sample sizes. The F1 score difference is also sensitive to the amount of overlapping percentage between the prior knowledge and target network (Figure 3E). When the overlapping percentage is larger (i.e., the prior information is more accurate), it provides more useful information for network reconstruction. In addition, we also performed simulations with *p* = 1000, *density* = 4*%*, *overlapping*(*ρ*) = 50*%* with similar results (data not shown).

### 3.3 AhGlasso Outperforms Conventional Methods

Recently, several groups have extended GGM to weighted Graphical Lasso (wGlasso) and network-based gene set analysis (Netgsa) to incorporate prior biological information (Table 1). These methods incorporate prior knowledge into graphical model-building procedures as well as gene set analysis. Recently, Yi *et al.* and Zuo *et al.* implemented weighted Graphical Lasso (Glasso) algorithms to incorporate prior known network information for network learning and the key difference between the two methods is how to select the *λ* parameter (Li and Jackson, 2015; Zuo et al., 2017). In Yi *et al*.’s study, *λ* was optimized by the BIC criteria (wGlasso_2015) while it was tuned with likelihood and cross-validation in Zuo *et* al.’s research (wGlasso_2017). Besides weighted Graphical Lasso, Ma *et al.* developed a Network-Based Gene Set analysis (Netgsa) approach for network learning and gene-set enrichment analysis by incorporating prior pathway information (Ma et al., 2016). The Netgsa approach combines the neighborhood selection technique (Meinshausen and Buhlmann, 2006) with constrained maximum likelihood estimation.

**TABLE 1:** Summary of network learning methods to incorporate prior knowledge.

Method	Published year	Abbreviation	Algorithm	Weight for prior knowledge	Model selection	Screening
Weighted Glasso (wGlasso)	2015	*wGlasso*_2015	Weighted Glasso	Continuous	BIC^a^	No
Weighted Glasso(wGlasso)	2017	wGlasso_2017	Weighted Glasso	Continuous	CV_log likelihood	No
Netgsa	2017	Netgsa	NB and Glasso	Binary	BIC^a^	No
AhGlasso	—	AhGlasso	NB and Glasso	Continuous	CV_BIC^b^	Yes

Notes: BIC, bayesian information criterion; CV, cross-validation; NB, neighbor selection; AhGlasso, augmented high-dimensional Graphical Lasso;

a , based on log Likelihood;

b , based on penalized log Likelihood.

**FIGURE 4:**
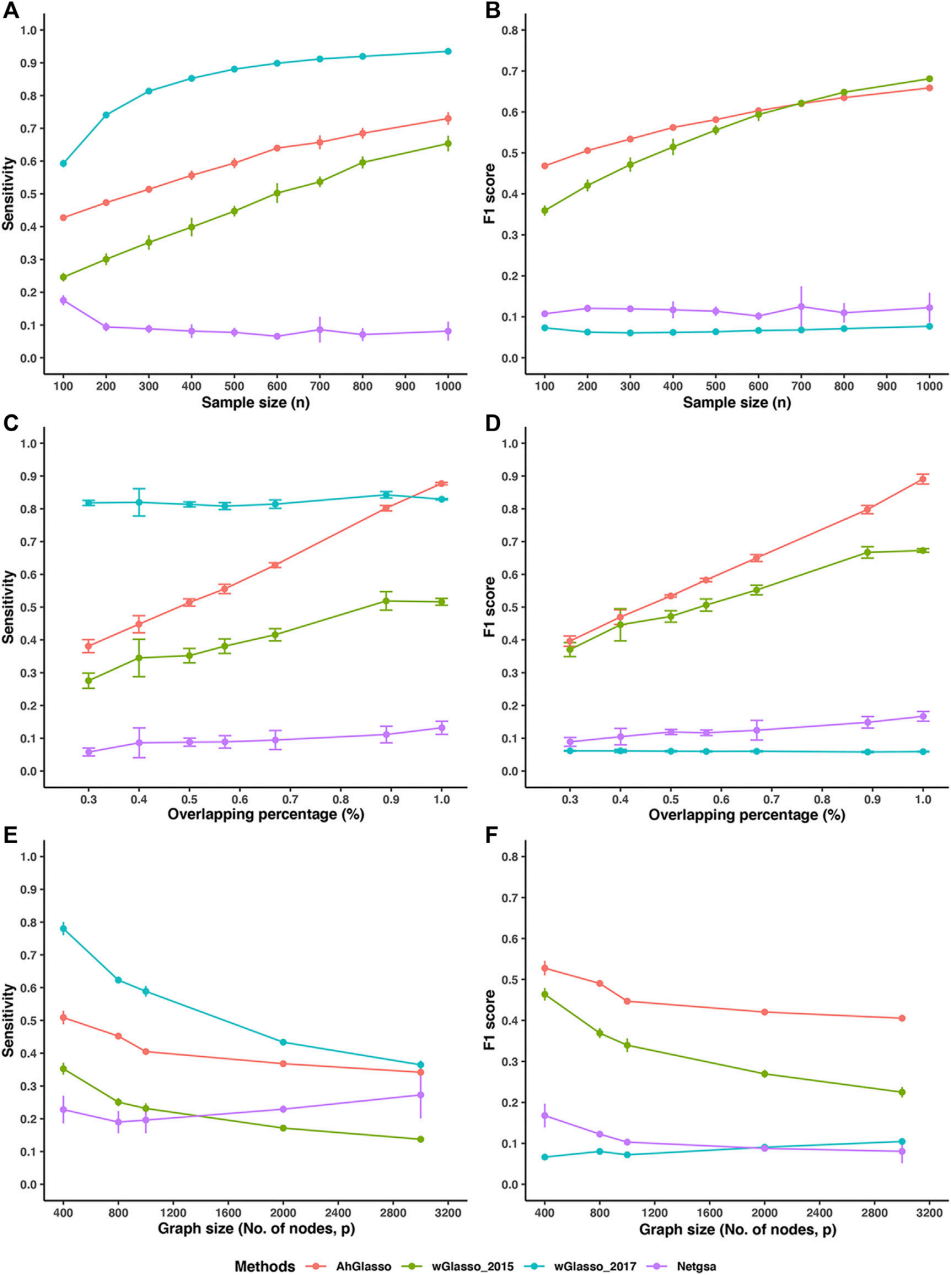
Method performance comparisons. The simulated protein network graph included 1000 (*p*) nodes. The density of the simulated graph is around 4*%*. **(A–B)** The overlapping between prior information and target true network is 50*%*. A variety of sample sizes of data was simulated as shown in X-axis. With the same true network and its corresponding covariance matrix (Σ_*true*_), we created various sizes (*n*) of multiple normal expression data for testing. We estimated the true network topology by using two weighted graphical Lasso (wGlasso_2015 and wGlasso_2017), Netgsa, and the proposed AhGlasso methods. The sensitivity **(A)** and F1 score **(B)** were calculated based on the estimated network and true network. **(C–D)** A variety of overlapping between prior information and target true network was simulated as shown in X-axis. The sample size was fixed at *n* = 300. The sensitivity **(C)** and F1 score **(D)** were calculated based on the estimated network and true network. **(E–F)** We investigated the effect of different graph sizes (p ranges from 400 to 3000) with a fixed sample size (*n* = 100) and the overlapping between prior network and target network (50*%*). The sensitivity **(E)** and F1 score **(F)** were calculated as previously. For each simulation setting, the simulations were repeated 5 times. The lines represent the mean scores for the simulated sample size and the error bars represent the standard error of the mean for each method.

We estimated the true network topology using AhGlasso and the three alternative methods (wGlasso_2015, wGlasso_2017, Netgsa). To make a fair comparison, we tuned the regularization parameter in each method with its designed optimization method. In this comparison study, we mimicked the high-dimensional setting and created simulation datasets with large *p* (1000) and various sample sizes. We found that our proposed method achieved higher sensitivity and F1 score than wGlasso_2015 when the sample size is small (Figure 4). They had similar performances when the sample size is large. These two methods achieved higher F1 scores and had an overall higher accuracy than the other two methods. The F1 score in the wGlasso_2017 method was consistently and extremely low although it achieved very high sensitivity. The specificity of wGlasso_2017 is very low (data not shown) since the weighted Graphical Lasso in Zuo *et a*l.’s study selects the model based on the log-likelihood only but does not penalize the number of edges, which leads to overfitting the model (over-dense output network). Although the Netgsa approach outperforms wGlasso_2017, its sensitivity and F1 scores are still substantially lower than our proposed method and wGlasso_2015. The MCC score patterns were similar to the F1 scores pattern (data not shown). Of note, the improvement of the proposed method decreases when compared with wGlasso_2015 when the sample size increases as shown in Figure 4B. However, it is not uncommon to have a limited sample size in high-throughput omics studies, especially in human studies using tissue samples but not blood.

We next investigated the effect of overlapping percentages between the prior network and target network with fixed graph size (*p* = 1000) and sample size (*n* = 300). We found that AhGlasso achieved higher sensitivity and F1 score than wGlasso_2015 and the difference increases when the overlapping percentage increases (Figures 4C,D). AhGlasso and wGlasso_2015 achieved higher F1 scores and had an overall higher accuracy than the other two methods.

In addition, we also investigated the effect of different graph sizes (p ranges from 400 to 3000) with a fixed sample size (*n* = 100) and the overlapping between prior network and target network (50*%*). We found that AhGlasso achieved higher sensitivity and F1 score than wGlasso_2015 and the differences increase when the graph size increases (Figures 4E,F). AhGlasso and wGlasso_2015 achieved higher F1 scores and had an overall higher accuracy than the other two methods (Figure 4F).

Since strong scale-free networks can be rare in biological contexts, we also compared the method performance on a simulated non-scale-free network. We first simulated a random network with *p* = 500 and different sample sizes ranging from 200 to 700. The overlapping percentage between prior knowledge and target network was set at 88*%*. We found that our proposed method has similar sensitivity scores to wGlasso_2015 and wGlasso_2017 (Supplementary Figure S1) and it outperforms the other three methods in terms of F1 scores in our tests (Supplementary Figure S1). In addition, we simulated with a fixed sample size (*n* = 300) but different degrees of overlapping between prior knowledge and target truth network. We also found that our proposed method outperforms the other three methods in terms of F1 scores in our tests (Supplementary Figure S2). Besides random networks, we also simulated networks with hub or cluster structures for comparison. We found that AhGlasso achieves higher F1 scores than wGlasso_2015, wGlasso_2017, and Netgsa in networks with both cluster network and hub network (data not shown).

**FIGURE 5:**
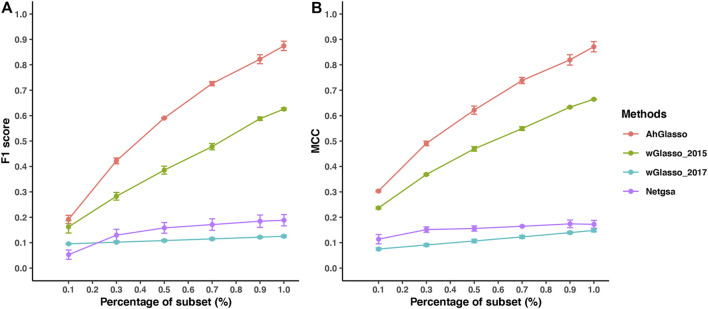
Method performance comparisons with partially known prior information. The simulated protein network graph includes 1000 (*p*) nodes with fixed sample size (*n* = 300). The density of the simulated graph is around 4*%*. The prior information is a subset of the target true network. A variety of subset percentages of the prior network were simulated as shown in X-axis. We estimated the true network topology by using two weighted graphical Lasso (wGlasso_2015 and wGlasso_2017), Netgsa, and the proposed AhGlasso methods. The F1 score **(A)** and MCC **(B)** were calculated based on the estimated network and true network. For each simulation setting, the simulations were repeated 5 times. The lines represent the mean scores for the simulated sample size and the error bars represent the standard error of the mean for each method.

In the real world, the prior PPI is often incomplete rather than purely noisy. We next investigated the method performance when the prior is a subset of the true network (i.e., incomplete prior information). We simulated datasets with a fixed sample size (*n* = 300) and graph density (4*%*). We randomly removed the connected edges in the prior network under the predefined subset percentage of the target network (Figure 5). When the subset percentage of the prior network increases, the F1 score increases in all the tested methods. Under the same subset percentage, our proposed AhGlasso method achieves a higher F1 score (Figure 5A) and MCC (Figure 5B) than the other three methods. The differences between AhGlasso and wGlasso_2015 increase when the subset percentage increases (Figure 5).

### 3.4 Comparison of Runtimes

**FIGURE 6:**
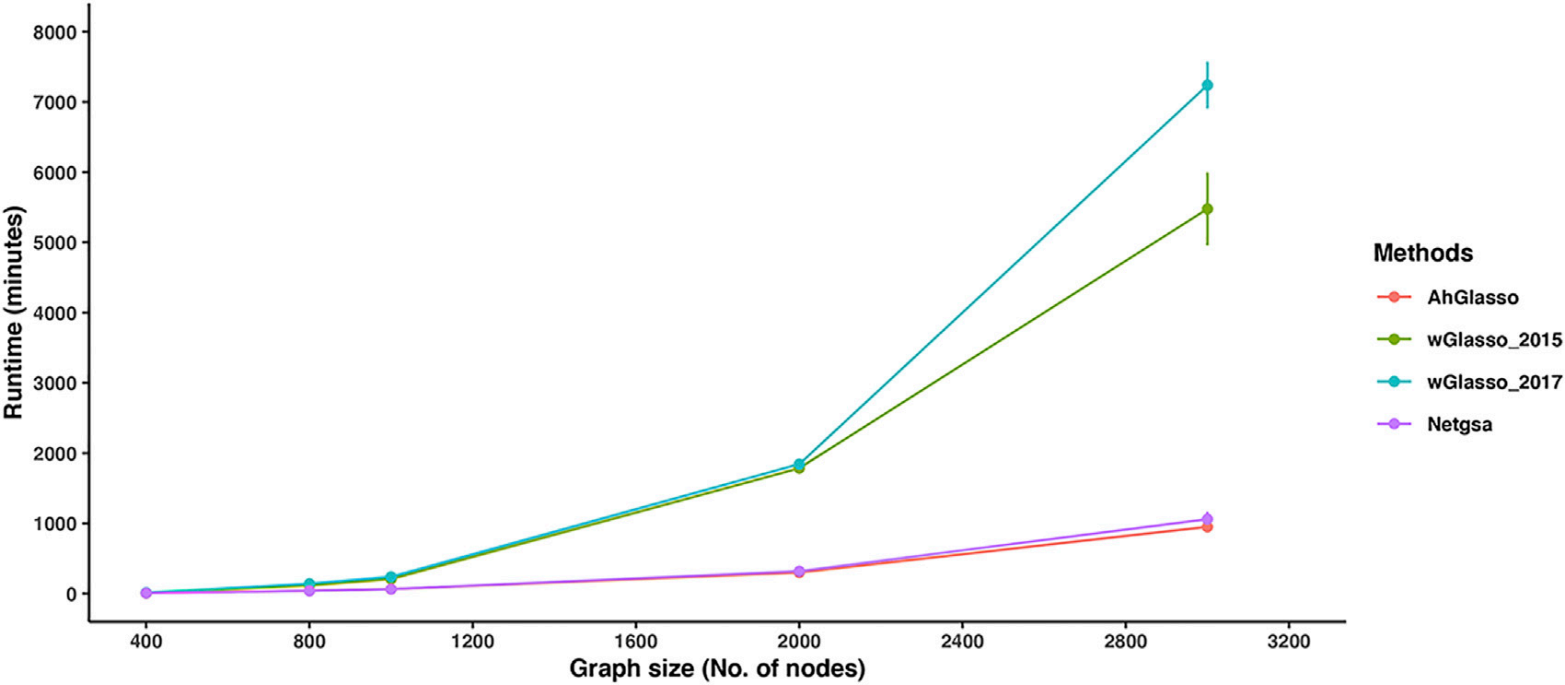
Method runtime comparisons. To compare the computational efficiency of the two weighted graphical Lasso (wGlasso_2015 and wGlasso_2017), Netgsa, and the proposed AhGlasso method in the high-dimensional setting, we simulated datasets with a fixed sample size (*n* = 200) and 4*%* graph density, but various graph size (*p* varies from 400 to 3000). We compared the total run time of parameter tuning (CV was removed if involved for fair comparisons) and the final model fitting with the chosen parameter. The *λ* minimal ratio was set to 0.01 and 40 *λ* candidates were searched. The simulation was repeated 5 times for each setting. The lines represent the mean runtime for the simulated graph size and the error bars represent the standard error of the mean for each method.

To compare the runtimes of the tested methods, we simulated datasets with a fixed sample size (*n* = 200) but various graph sizes (*p* varies from 400 to 3000). We evaluated the computation time based on an Intel(R) Xeon(R) Gold 6152 CPU @ 2.10 GHz CentOS Linux 7 (Core) operating system. Since the regularization parameter tuning in each method is a critical step, we compare the total run time of parameter tuning (CV was removed if involved for fair comparisons) and the final model fitting with the chosen parameter. The simulation was repeated 5 times for each setting. With *p* = 1000, the total running time for AhGlasso, wGasso_2015, wGlasso_2017, and Netgsa was 64 ± 6.64 min, 207.51 ± 36.70 min, 239.97 ± 44.28 min, and 64.34 ± 7.4 min, respectively (Figure 6). The runtimes of AhGlasso and Netgsa are comparable and they are around 4 times faster than the two weighted Graphical Lasso-based algorithms when *p* = 1000. The runtimes of wGlasso_2015 and wGlasso_2017 exponentially increase when the graph size increases. With *p* = 3000, the total running time for AhGlasso, wGasso_2015, wGlasso_2017, and Netgsa was 951.09 ± 173.51 min, 5477.11 ± 815.34 min, 7238.65 ± 523.54 min, and 1060.65 ± 238.83 min, respectively. The runtime of AhGlasso is around 7 times faster than the two weighted Graphical Lasso-based algorithms when *p* = 3000. The runtime of AhGlasso is also faster than Netgsa. Of note, the F1 score patterns in these simulations for tested methods were similar to Figure 4B (data not shown). In addition, the runtimes of weighted Graphical Lasso-based algorithms are much more sensitive to the *λ* minimal ratio than AhGlasso and Netgsa. The runtime differences between AhGlasso and weighted Graphical Lasso-based algorithms are greater when decreasing the *λ* minimal ratio and searching more *λ* candidates.

When the feature space is large, the graphical Lasso-based methods are computationally expensive and even intractable. AhGlasso preselects the potential neighbor nodes with *ψ* screening to narrow down the neighbor node space. In addition, the Meinshausen-Bühlmann algorithm (Meinshausen and Buhlmann, 2006) is incorporated in AhGlasso to select neighbor nodes before maximum likelihood estimation. Due to the screening and selection steps, AhGlasso is more efficient and faster than weighted Glasso methods in large-scale graph construction.

### 3.5 AhGlasso Improves Network Inference for COPD

For a real data application, we used proteomics data generated in the COPDGene Phase II Study. COPD is a disease characterized by reduced lung function and symptoms such as shortness of breath. Protein-protein interactions could play important roles in COPD pathogenesis in COPD development. We collected a large proteomic data from 1010 subjects from the COPDgene cohort using the SomaScan® platform (Candia et al., 2017). We constructed a COPD-associated network on COPD cases (*n* = 486) with or without PPI data and identified important protein-protein interactions contributing to COPD development.

We incorporated prior known PPI information from STRING and constructed COPD-associated networks with the AhGlasso and Netgsa methods. The top 40 hub proteins were chosen for GO enrichment analysis. In the AhGlasso analysis with known PPI knowledge, we found 35 molecular function pathways that were significantly enriched while only 12 pathways were enriched in the analysis without prior PPI knowledge (Supplementary Table S1 and Supplementary Table S1), and only 17 pathways were enriched in the Netgsa analysis (Supplementary Table S1). After multiple testing corrections with FDR, we found 23 molecular function pathways that were significantly enriched in AhGlasso analysis with prior PPI information while only one pathway was enriched in the analysis without prior PPI knowledge, and no pathways were enriched in the Netgsa analysis. AhGlasso also identified six hub genes related to the cadherin pathway, which has been reported to play important roles in COPD development (Nelson and Nusse, 2004; Kneidinger et al., 2011; Eapen and Sohal, 2020), but was not enriched in the results of the Netgsa method. In addition, AhGlasso also uniquely enriched cytokine signaling and chemokine signaling pathways in COPD-associated networks, which have been reported to be important for COPD pathogenesis (Bradford et al., 2017; Henrot et al., 2019).

## 4 Discussion

In this study, we have developed an augmented high-dimensional Graphical Lasso model (AhGlasso) to incorporate edge weights from known protein-protein interaction networks with omics data for global network learning. In our proposed method, we first extended the Netgsa hybrid approach to incorporate the edge weight of prior known but incomplete protein-protein relation for network reconstruction. To speed up the computation and make it feasible for large-scale data, especially when the number of variables is much larger than the sample size, we also implemented Ψ-screening based on the standard Pearson correlation. We compared our proposed method with Netgsa and two weighted Graphical Lasso approaches in terms of computation time and accuracy based on simulations where “ground truth” for the target network is available.

To systematically evaluate the performance of methods and make fair comparisons, we simulated datasets with a variety of network graph sizes, sample sizes, and overlapping percentages between the prior information and the target network for estimation. For network structure learning with GMM, the *λ* parameter controls the sparsity of the output network. Tuning the *λ* parameter is a key step for AhGlasso and other Lasso-based approaches. However, there is no consensus on how to select the *λ* regularization parameter. Cross-validation (CV) provides a general tool to solve this kind of challenge. Our simulation study suggested that cross-validation with BIC is more stable and outperforms the other criteria when the sample size is medium. When the sample size is small (such as *n* < (0.25 × *p*)), conventional BIC has similar or even better performance than BIC with cross-validation, which may be explained that the small sample sizes for the K fold cross-validation lead to large variance. When the sample size is very large, the performance of conventional BIC and BIC with cross-validation are similar, which could be explained that the models with or without cross-validation converge to the unbiased true model when data is sufficient. In addition, we also found that cross-validation with AIC and EBIC often results in the under-fitting of the model and over-sparse networks, leading to lower F1 score and MCC.

Our simulation study found that the AhGlasso output estimated networks with *a priori* information had a significantly greater sensitivity, F1 score, MCC than the estimations without *a priori* information. The F1 score difference decreases for larger sample sizes because the impact of incorporating prior information decreases with more data. The F1 score difference is also sensitive to the amount of overlapping percentage between the prior knowledge and the target network. As expected, when the prior information is less accurate, it provides less useful information for network reconstruction. However, when the overlapping percentage between prior information and the target network is low, we did not observe an increase in the false-positive rate.

The key difference between the two weighted Glasso methods that incorporate prior known network information for network graph learning is how they select the *λ* parameter (Li and Jackson, 2015; Zuo et al., 2017). In wGlasso_2015, the *λ* was optimized with the BIC criteria while it was tuned with likelihood and cross-validation in wGlasso_2017 (Li and Jackson, 2015; Zuo et al., 2017). The wGlasso_2017 approach often resulted in high sensitivity but extremely low specificity, which leads to a low F1 score. This could be explained that wGlasso_2017 tunes the *λ* parameter based on likelihood with a lack of penalty for the number of edges, which leads to an over-dense network estimation. Our study also demonstrated AhGlasso generally outperforms Netgsa in terms of F1 scores, which suggests the advantages of taking edge weights into account. Although wGlasso_2015 could achieve comparable accuracy to AhGlasso, its computation is intractable for large-scale data. It took days when the network graph size is bigger than 1000. Compared with wGlasso_2015, our proposed method, AhGlasso, is computationally scalable and much more efficient than the weighted Glasso based methods when the sample size is large. In summary, the new method, AhGlasso, outperforms wGlasso-based algorithms with respect to computational time in simulated large-scale data settings, while achieving better or comparable prediction accuracy of node connections.

In the COPDgene study, AhGlasso with prior PPI found more enriched GO terms than the estimated network without PPI on the top 40 hub proteins in the resultant networks (23 vs. 1 after multiple testing correction with FDR). We also found AhGlasso discovered more enriched GO terms than Netgsa on the top 40 hub proteins in the resultant networks (23 vs. 0 after FDR correction). After FDR correction, we found significantly enriched cadherin, chemokine signaling, cytokine signaling pathways in AhGlasso, but not with the Netgsa analysis or the analysis without PPI. These three pathways have been demonstrated to play important roles in COPD. Several studies reported that the cadherin/WNT/catenin pathway could be a novel therapeutic target for attenuating airway remodeling in COPD (Nelson and Nusse, 2004; Kneidinger et al., 2011; Eapen and Sohal, 2020). This real data study suggests that AhGlasso improves COPD-related network inference compared to the Netgsa approach in integrating a proteomics dataset and prior PPI with edge weights.

Although we only focused on analyzing single omics data in this study, the partial correlation coefficient derived in AhGlasso can be transformed to a *Z* score via Fisher’s transformation for multiple omics data integration. The *Z* scores from different single omics data can be easily combined using Stouffer’s meta-analysis method (Stouffer et al., 1949).

Although AhGlasso outperforms wGlasso-based algorithms and Netgsa in our simulations and COPD case study, there are some limitations in the method. One limitation is that AhGlasso can be computationally intensive when the graph size is large, but still faster than the alternatives compared. Another limitation is that the optional K-fold cross-validation step can significantly increase the computation burden. Furthermore, K-fold cross-validation is not recommended if the sample size is small. Although we compared AhGlasso with wGlasso_2017 and Netgsa, the latter were not designed for global network learning. The wGlasso_2017 approach is designed for network-based differential gene expression analysis using differentially weighted graphical Lasso on pre-selected differentially expressed genes. The Netgsa approach is designed for network-based pathway enrichment analysis and focused on protein-protein interaction changes in known pathways. Furthermore, to implement Netgsa non-zero weights in the prior PPI were converted to 1, which could cause potential biases.

## 5 Conclusion

We present an augmented method, called AhGlasso, for incorporating prior information in biological network reconstruction. Our study suggests that cross-validation with BIC generally performs better than regular BIC, cross-validation AIC, or EBIC. This new method outperforms wGlasso-based algorithms with respect to computational time in large-scale data settings while achieving better or comparable prediction accuracy of edges. Our method also improves COPD-associated network inference compared to the Netgsa approach. Although demonstrated on one -omics data and prior PPI, our method could be generalized to multi-omics data.

## Data Availability

Clinical Data and SOMAScan data in this study are available through COPDGene (https://www.ncbi.nlm.nih.gov/gap/, ID: phs000179. v6. p2).
