# Supplementary material for: An Augmented High-Dimensional Graphical Lasso Method to Incorporate Prior Biological Knowledge for Global Network Learning
Source: Front Genet. 2022 Jan 27;12:760299. doi: 10.3389/fgene.2021.760299 (PMC8829118; doi:10.3389/fgene.2021.760299)
Supplement: Supplementary file 1 [file DataSheet1.pdf]

Supplementary Material

1 SUPPLEMENTARY METHOD

1.1 Ψ-screening and Ψ partial correlation coefficient

In Liang *et al.*'s study, the partial correlation coefficient Ψ_{ik} was defined by

$$\Psi_{ik} = \{\psi_{ik}\}, \tag{S1}$$

where $\psi_{ik} = \hat{\varepsilon}_{i,-k}$ if $|\hat{\varepsilon}_{i,-k}| < |\hat{\varepsilon}_{k,-i}|$ and $\psi_{ik} = \hat{\varepsilon}_{k,-i}$ otherwise. With the partial correlation coefficients, the network structure could be learned with the following Ψ algorithm proposed in the previous study (Liang et al., 2015):

Step 1, Correlation screening: Determine the reduced neighborhood for each variable $X(i)$;

a) Conduct a multiple hypothesis test to identify the pairs of vertices for which the empirical correlation coefficient is significantly different from zero (empirical correlation network);

b) For each variable $X(i)$, identify its neighborhood in the empirical correlation network, and reduce the size of the neighborhood by removing the variables having a lower correlation (in absolute value).

Step 2, Ψ-calculation: For each pair of vertices i and j, identify the separator S_{ij} based on the reduced correlation network resulted in step (1) and calculate Ψ_{ij} by inverting the subsample covariance matrix;

Step 3, Ψ-screening: Conduct a multiple hypothesis test to identify the pairs of vertices for which Ψ_{ij} is significantly different from zero. If the pairs of vertices are not significantly different from 0, these edges were set to 0 to reduce dimensionality.

Similar to the *huge* R library, we adapted the correlation screening step in Ψ-algorithem to AhGlasso to reduce the size of potential neighborhood and speed up the estimation.

2 SUPPLEMENTARY TABLES AND FIGURES

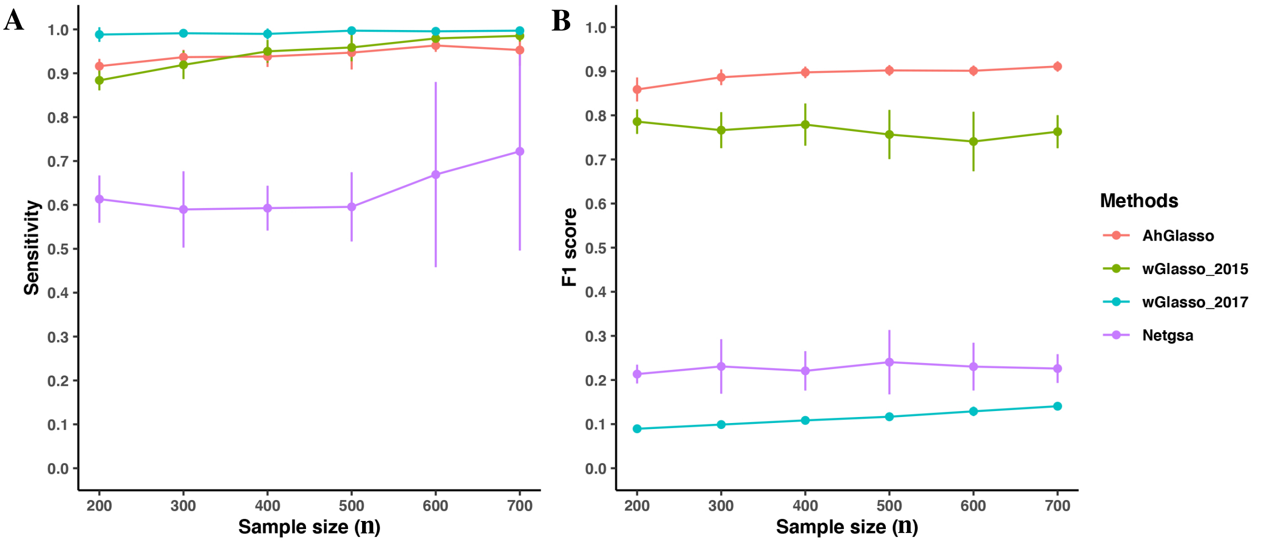

Figure S1. Method performance comparisons in a non-scale free random network. The simulated random network graph included 500 (p) nodes. The overlap between prior information and target true network is 88%. With the same true network and its corresponding covariance matrix (Σ_{true}), we created various sizes (n) of multiple normal expression data for testing. We estimated the true network topology by using two weighted graphical LASSO (wGlasso_2015 and wGlasso_2017), Netgsa, and the proposed AhGlasso method. The λ was optimized with each designed criteria as shown in Table 1. The F1 score and MCC were calculated based on the estimated network and true network. For each simulation setting, the simulations were repeated 5 times. The lines represent the mean scores for the simulated sample size and the error bars represent the standard error of the mean for each method. Of note, similar results were achieved in various p and n simulations.

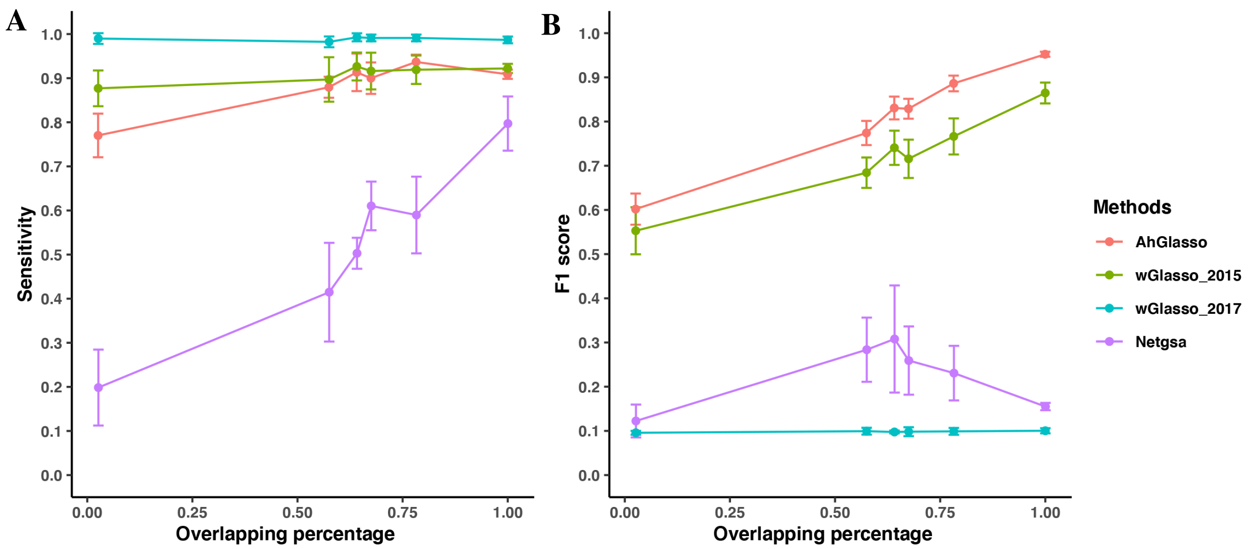

Figure S2. Method performance comparisons in a non-scale free random network. The simulated random network graph included 500 (p) nodes. The overlap between prior information and target true network varied as indicated. With the same true network and its corresponding covariance matrix (Σ_{true}), we created multiple normal expression data for testing with $n = 300$. We estimated the true network topology by using two weighted graphical LASSO (wGlasso_2015 and wGlasso_2017), Netgsa, and the proposed AhGlasso method. The λ was optimized with each designed criteria as shown in Table 1. The F1 score and MCC were calculated based on the estimated network and true network. For each simulation setting, the simulations were repeated 5 times. The lines represent the mean scores for the simulated sample size and the error bars represent the standard error of the mean for each method.

Table S1. GO enrichment of the top 40 hub proteins in estimated network without prior PPI knowledge.

	GO.ID	Term	Annotated	Significant	Expected	P value	Adjusted P value
1	GO:0008047	enzyme activator activity	48	7	1.47	0.00041	**0.0455**
2	GO:0019899	enzyme binding	251	15	7.7	0.00421	0.182
3	GO:0003676	nucleic acid binding	136	10	4.17	0.0056	0.182
4	GO:0070851	growth factor receptor binding	76	7	2.33	0.00656	0.182
5	GO:0045296	cadherin binding	47	5	1.44	0.01227	0.2445
6	GO:0030234	enzyme regulator activity	132	9	4.05	0.01449	0.2445
7	GO:0003723	RNA binding	89	7	2.73	0.01542	0.2445
8	GO:0008009	chemokine activity	36	4	1.1	0.02178	0.3022
9	GO:0042379	chemokine receptor binding	39	4	1.2	0.02848	0.3513
10	GO:0031625	ubiquitin protein ligase binding	42	4	1.29	0.0363	0.3587
11	GO:0030546	signaling receptor activator activity	213	11	6.53	0.04677	0.3587
12	GO:0044389	ubiquitin-like protein ligase binding	46	4	1.41	0.0485	0.3587

Note:

Annotated, number of proteins in a pathway from the complete set of 1212 proteins;

Significant, number of protens in a pathway from 40 hub proteins;

Expected, the expected number of proteins in a pathway if we randomly selected 40 proteins from 1212 background proteins;

P value: Fisher'sexact test

Adjusted P value: Benjamini-Hochberg adjusted P value to control for False Discover Rate

Table S2. GO enrichment of the top 40 hub proteins in AhGlasso estimated network.

	GO.ID	Term	Annotated	Significant	Expected	P value	Adjuted P value
1	GO:0005102	signaling receptor binding	427	29	14.55	2.50E-06	3.00E-04
2	GO:0042802	identical protein binding	251	21	8.55	1.00E-05	6.00E-04
3	GO:0005178	integrin binding	49	8	1.67	0.00014	0.0042
4	GO:0098772	molecular function regulator	370	24	12.61	0.00015	0.0042
5	GO:0050839	cell adhesion molecule binding	115	12	3.92	0.00023	0.0051
6	GO:0001664	G protein-coupled receptor binding	68	9	2.32	0.00028	0.0051
7	GO:0044877	protein-containing complex binding	197	16	6.71	0.00032	0.0051
8	GO:0005126	cytokine receptor binding	130	12	4.43	0.00075	0.0097
9	GO:0002020	protease binding	35	6	1.19	0.00083	0.0097
10	GO:0030234	enzyme regulator activity	132	12	4.5	0.00087	0.0097
11	GO:0003677	DNA binding	68	8	2.32	0.00145	0.0146
12	GO:0019899	enzyme binding	251	17	8.55	0.00179	0.0166
13	GO:0019904	protein domain specific binding	73	8	2.49	0.00232	0.0198
14	GO:0045296	cadherin binding	47	6	1.6	0.00406	0.0322
15	GO:0000976	transcription regulatory region sequence...	35	5	1.19	0.00544	0.0377
16	GO:0001067	regulatory region nucleic acid binding	35	5	1.19	0.00544	0.0377
17	GO:0048018	receptor ligand activity	210	14	7.16	0.00647	0.0421
18	GO:0005125	cytokine activity	125	10	4.26	0.00683	0.0421
19	GO:0030546	signaling receptor activator activity	213	14	7.26	0.00738	0.0421
20	GO:0008083	growth factor activity	88	8	3	0.00759	0.0421
21	GO:0042379	chemokine receptor binding	39	5	1.33	0.00872	0.044
22	GO:1990837	sequence-specific double-stranded DNA bi...	39	5	1.33	0.00872	0.044
23	GO:0030545	receptor regulator activity	221	14	7.53	0.01034	0.0499
24	GO:0070851	growth factor receptor binding	76	7	2.59	0.01187	0.053
25	GO:0043565	sequence-specific DNA binding	42	5	1.43	0.01194	0.053
26	GO:0003690	double-stranded DNA binding	43	5	1.47	0.01318	0.0542
27	GO:0140110	transcription regulator activity	43	5	1.47	0.01318	0.0542
28	GO:0140297	DNA-binding transcription factor binding	30	4	1.02	0.01669	0.0662
29	GO:0044389	ubiquitin-like protein ligase binding	46	5	1.57	0.0174	0.0666
30	GO:0008134	transcription factor binding	50	5	1.7	0.02432	0.09
31	GO:0008270	zinc ion binding	73	6	2.49	0.03325	0.1139
32	GO:1901363	heterocyclic compound binding	304	16	10.36	0.03327	0.1139
33	GO:0003676	nucleic acid binding	136	9	4.63	0.03387	0.1139
34	GO:0097159	organic cyclic compound binding	311	16	10.6	0.04068	0.1328
35	GO:0019902	phosphatase binding	41	4	1.4	0.04709	0.1493

Note:

Annotated, number of proteins in a pathway from the complete set of 1212 proteins;

Significant, number of protens in a pathway from 40 hub proteins;

Expected, the expected number of proteins in a pathway if we randomly selected 40 proteins from 1212 background proteins;

P value: Fisher's exact test

Adjusted P value: Benjamini-Hochberg adjusted P value to control for False Discovery Rate

Table S3. GO enrichment of the top 40 hub proteins in Netgsa estimated network.

	GO.ID	Term	Annotated	Significant	Expected	P value	adjusted P value
1	GO:0002020	protease binding	35	6	1.19	0.00083	0.0921
2	GO:0042802	identical protein binding	251	17	8.55	0.00179	0.0993
3	GO:0004866	endopeptidase inhibitor activity	56	6	1.91	0.00977	0.1329
4	GO:0005539	glycosaminoglycan binding	93	8	3.17	0.01059	0.1329
5	GO:0019838	growth factor binding	57	6	1.94	0.01064	0.1329
6	GO:0030414	peptidase inhibitor activity	57	6	1.94	0.01064	0.1329
7	GO:0005102	signaling receptor binding	427	22	14.55	0.01117	0.1329
8	GO:0061135	endopeptidase regulator activity	58	6	1.98	0.01157	0.1329
9	GO:0031625	ubiquitin protein ligase binding	42	5	1.43	0.01194	0.1329
10	GO:0004857	enzyme inhibitor activity	77	7	2.62	0.01272	0.1329
11	GO:0019899	enzyme binding	251	15	8.55	0.01317	0.1329
12	GO:0061134	peptidase regulator activity	63	6	2.15	0.01711	0.1486
13	GO:0044389	ubiquitin-like protein ligase binding	46	5	1.57	0.0174	0.1486
14	GO:0005201	extracellular matrix structural constitu...	33	4	1.12	0.02315	0.1835
15	GO:0008201	heparin binding	69	6	2.35	0.02592	0.1918
16	GO:0019904	protein domain specific binding	73	6	2.49	0.03325	0.2307
17	GO:1901681	sulfur compound binding	80	6	2.73	0.04914	0.3209

Note:
Annotated, number of proteins in a pathway from the complete set of 1212 proteins;
Significant, number of protens in a pathway from 40 hub proteins;
Expected, the expected number of proteins in a pathway if we randomly selected 40 proteins from 1212 background proteins;
P value: Fisher's exact test
Adjusted P value: Benjamini-Hochberg adjusted P value to control for False Discovery Rate

3 SUPPLEMENTARY ACKNOWLEDGE

COPDGene Phase 3

Grant Support and Disclaimer

The project described was supported by Award Number U01 HL089897 and Award Number U01 HL089856 from the National Heart, Lung, and Blood Institute. The content is solely the responsibility of the authors and does not necessarily represent the official views of the National Heart, Lung, and Blood Institute or the National Institutes of Health.

COPD Foundation Funding

COPDGene is also supported by the COPD Foundation through contributions made to an Industry Advisory Board that has included AstraZeneca, Bayer Pharmaceuticals, Boehringer-Ingelheim, Genentech, GlaxoSmithKline, Novartis, Pfizer, and Sunovion.

COPDGene® Investigators – Core Units

Administrative Center: James D. Crapo, MD (PI); Edwin K. Silverman, MD, PhD (PI); Barry J. Make, MD; Elizabeth A. Regan, MD, PhD

Genetic Analysis Center: Terri H. Beaty, PhD; Peter J. Castaldi, MD, MSc; Michael H. Cho, MD, MPH; Dawn L. DeMeo, MD, MPH; Adel El Boueiz, MD, MMSc; Marilyn G. Foreman, MD, MS; Auyon Ghosh, MD; Lystra P. Hayden, MD, MMSc; Craig P. Hersh, MD, MPH; Jacqueline Hetmanski, MS; Brian D. Hobbs, MD, MMSc; John E. Hokanson, MPH, PhD; Wonji Kim, PhD; Nan Laird, PhD; Christoph Lange, PhD; Sharon M. Lutz, PhD; Merry-Lynn McDonald, PhD; Dmitry Prokopenko, PhD; Matthew Moll, MD, MPH; Jarrett Morrow, PhD; Dandi Qiao, PhD; Elizabeth A. Regan, MD, PhD; Aabida Saferali, PhD; Phuwanat Sakornsakolpat, MD; Edwin K. Silverman, MD, PhD; Emily S. Wan, MD; Jeong Yun, MD, MPH

Imaging Center: Juan Pablo Centeno; Jean-Paul Charbonnier, PhD; Harvey O. Coxson, PhD; Craig J. Galban, PhD; MeiLan K. Han, MD, MS; Eric A. Hoffman, Stephen Humphries, PhD; Francine L. Jacobson, MD, MPH; Philip F. Judy, PhD; Ella A. Kazerooni, MD; Alex Kluiber; David A. Lynch, MB; Pietro Nardelli, PhD; John D. Newell, Jr., MD; Aleena Notary; Andrea Oh, MD; Elizabeth A. Regan, MD, PhD; James C. Ross, PhD; Raul San Jose Estepar, PhD; Joyce Schroeder, MD; Jered Sieren; Berend C. Stoel, PhD; Juerg Tschirren, PhD; Edwin Van Beek, MD, PhD; Bram van Ginneken, PhD; Eva van Rikxoort, PhD; Gonzalo Vegas Sanchez-Ferrero, PhD; Lucas Veitel; George R. Washko, MD; Carla G. Wilson, MS;

PFT QA Center, Salt Lake City, UT: Robert Jensen, PhD

Data Coordinating Center and Biostatistics, *National Jewish Health, Denver, CO*: Douglas Everett, PhD; Jim Crooks, PhD; Katherine Pratte, PhD; Matt Strand, PhD; Carla G. Wilson, MS

Epidemiology Core, *University of Colorado Anschutz Medical Campus, Aurora, CO*: John E. Hokanson, MPH, PhD; Erin Austin, PhD; Gregory Kinney, MPH, PhD; Sharon M. Lutz, PhD; Kendra A. Young, PhD

Version Date: March 26, 2021

Mortality Adjudication Core: Surya P. Bhatt, MD; Jessica Bon, MD; Alejandro A. Diaz, MD, MPH; MeiLan K. Han, MD, MS; Barry Make, MD; Susan Murray, ScD; Elizabeth Regan, MD; Xavier Soler, MD; Carla G. Wilson, MS

Biomarker Core: Russell P. Bowler, MD, PhD; Katerina Kechris, PhD; Farnoush Banaei-Kashani, PhD

COPDGene® Investigators – Clinical Centers

Ann Arbor VA: Jeffrey L. Curtis, MD; Perry G. Pernicano, MD

Baylor College of Medicine, Houston, TX: Nicola Hanania, MD, MS; Mustafa Atik, MD; Aladin Boriek, PhD; Kalpatha Guntupalli, MD; Elizabeth Guy, MD; Amit Parulekar, MD;

Brigham and Women's Hospital, Boston, MA: Dawn L. DeMeo, MD, MPH; Craig Hersh, MD, MPH; Francine L. Jacobson, MD, MPH; George Washko, MD

Columbia University, New York, NY: R. Graham Barr, MD, DrPH; John Austin, MD; Belinda D'Souza, MD; Byron Thomashow, MD

Duke University Medical Center, Durham, NC: Neil MacIntyre, Jr., MD; H. Page McAdams, MD; Lacey Washington, MD

HealthPartners Research Institute, Minneapolis, MN: Charlene McEvoy, MD, MPH; Joseph Tashjian, MD

Johns Hopkins University, Baltimore, MD: Robert Wise, MD; Robert Brown, MD; Nadia N. Hansel, MD, MPH; Karen Horton, MD; Allison Lambert, MD, MHS; Nirupama Putcha, MD, MHS

Lundquist Institute for Biomedical Innovation at Harbor UCLA Medical Center, Torrance, CA: Richard Casaburi, PhD, MD; Alessandra Adami, PhD; Matthew Budoff, MD; Hans Fischer, MD; Janos Porszasz, MD, PhD; Harry Rossiter, PhD; William Stringer, MD

Michael E. DeBakey VAMC, Houston, *TX*: Amir Sharafkhaneh, MD, PhD; Charlie Lan, DO

Minneapolis VA: Christine Wendt, MD; Brian Bell, MD; Ken M. Kunisaki, MD, MS

Morehouse School of Medicine, Atlanta, GA: Eric L. Flenaugh, MD; Hirut Gebrekristos, PhD; Mario Ponce, MD; Silanath Terpenning, MD; Gloria Westney, MD, MS

National Jewish Health, Denver, CO: Russell Bowler, MD, PhD; David A. Lynch, MB

Reliant Medical Group, Worcester, MA: Richard Rosiello, MD; David Pace, MD

Temple University, Philadelphia, PA: Gerard Criner, MD; David Ciccolella, MD; Francis Cordova, MD; Chandra Dass, MD; Gilbert D'Alonzo, DO; Parag Desai, MD; Michael Jacobs, PharmD; Steven Kelsen, MD, PhD; Victor Kim, MD; A. James Mamary, MD; Nathaniel

Version Date: March 26, 2021

Marchetti, DO; Aditi Satti, MD; Kartik Shenoy, MD; Robert M. Steiner, MD; Alex Swift, MD; Irene Swift, MD; Maria Elena Vega-Sanchez, MD

University of Alabama, Birmingham, AL: Mark Dransfield, MD; William Bailey, MD; Surya P. Bhatt, MD; Anand Iyer, MD; Hrudaya Nath, MD; J. Michael Wells, MD

University of California, San Diego, CA: Douglas Conrad, MD; Xavier Soler, MD, PhD; Andrew Yen, MD

University of Iowa, Iowa City, IA: Alejandro P. Comellas, MD; Karin F. Hoth, PhD; John Newell, Jr., MD; Brad Thompson, MD

University of Michigan, Ann Arbor, MI: MeiLan K. Han, MD MS; Ella Kazerooni, MD MS; Wassim Labaki, MD MS; Craig Galban, PhD; Dharshan Vummidi, MD

University of Minnesota, Minneapolis, MN: Joanne Billings, MD; Abbie Begnaud, MD; Tadashi Allen, MD

University of Pittsburgh, Pittsburgh, PA: Frank Sciurba, MD; Jessica Bon, MD; Divay Chandra, MD, MSc; Joel Weissfeld, MD, MPH

University of Texas Health, San Antonio, San Antonio, TX: Antonio Anzueto, MD; Sandra Adams, MD; Diego Maselli-Caceres, MD; Mario E. Ruiz, MD; Harjinder Singh

Version Date: March 26, 2021

REFERENCES

Liang, F., Song, Q., and Qiu, P. (2015). An equivalent measure of partial correlation coefficients for high-dimensional gaussian graphical models. *Journal of the American Statistical Association* 110, 1248–1265
