# Supplementary material for: An Augmented High-Dimensional Graphical Lasso Method to Incorporate Prior Biological Knowledge for Global Network Learning
Source: Front Genet. 2022 Jan 27;12:760299. doi: 10.3389/fgene.2021.760299 (PMC8829118; doi:10.3389/fgene.2021.760299)
Supplement: Supplementary file 2 [file DataSheet2.ZIP › Frontiers_LaTex_AhGlasso/ahGlasso_topGo_40.pdf]

Table S1. GO enrichment of the top 40 hub proteins in AhGlasso estimated network.

	GO.ID	Term	Annotated	Significant	Expected	P value
1	GO:0005178	integrin binding	49	8	1.67	0.00014
2	GO:0098772	molecular function regulator	370	24	12.61	0.00015
3	GO:0050839	cell adhesion molecule binding	115	12	3.92	0.00023
4	GO:0001664	G protein-coupled receptor binding	68	9	2.32	0.00028
5	GO:0044877	protein-containing complex binding	197	16	6.71	0.00032
6	GO:0005126	cytokine receptor binding	130	12	4.43	0.00075
7	GO:0002020	protease binding	35	6	1.19	0.00083
8	GO:0030234	enzyme regulator activity	132	12	4.5	0.00087
9	GO:0003677	DNA binding	68	8	2.32	0.00145
10	GO:0019899	enzyme binding	251	17	8.55	0.00179
11	GO:0019904	protein domain specific binding	73	8	2.49	0.00232
12	GO:0045296	cadherin binding	47	6	1.6	0.00406
13	GO:0000976	transcription regulatory region sequence...	35	5	1.19	0.00544
14	GO:0001067	regulatory region nucleic acid binding	35	5	1.19	0.00544
15	GO:0048018	receptor ligand activity	210	14	7.16	0.00647
16	GO:0005125	cytokine activity	125	10	4.26	0.00683
17	GO:0030546	signaling receptor activator activity	213	14	7.26	0.00738
18	GO:0008083	growth factor activity	88	8	3	0.00759
19	GO:0042379	chemokine receptor binding	39	5	1.33	0.00872
20	GO:1990837	sequence-specific double-stranded DNA bi...	39	5	1.33	0.00872
21	GO:0030545	receptor regulator activity	221	14	7.53	0.01034
22	GO:0070851	growth factor receptor binding	76	7	2.59	0.01187
23	GO:0043565	sequence-specific DNA binding	42	5	1.43	0.01194
24	GO:0003690	double-stranded DNA binding	43	5	1.47	0.01318
25	GO:0140110	transcription regulator activity	43	5	1.47	0.01318
26	GO:0140297	DNA-binding transcription factor binding	30	4	1.02	0.01669
27	GO:0044389	ubiquitin-like protein ligase binding	46	5	1.57	0.0174
28	GO:0008134	transcription factor binding	50	5	1.7	0.02432
29	GO:0008270	zinc ion binding	73	6	2.49	0.03325
30	GO:1901363	heterocyclic compound binding	304	16	10.36	0.03327
31	GO:0003676	nucleic acid binding	136	9	4.63	0.03387
32	GO:0097159	organic cyclic compound binding	311	16	10.6	0.04068
33	GO:0019902	phosphatase binding	41	4	1.4	0.04709

Note:

Annoted, the number of proteins in a pathway within 1212 proteins;

Significant, the number of protens in a pathway within 40 hub proteins;

Expected, the expected number of proteins in a pathway if we randomly selected 40 proteins from 1212 background proteins;

P value: Fisher's test
