# Supplementary material for: An Augmented High-Dimensional Graphical Lasso Method to Incorporate Prior Biological Knowledge for Global Network Learning
Source: Front Genet. 2022 Jan 27;12:760299. doi: 10.3389/fgene.2021.760299 (PMC8829118; doi:10.3389/fgene.2021.760299)
Supplement: Supplementary file 2 [file DataSheet2.ZIP › Frontiers_LaTex_AhGlasso/netgsa_topGo_40.pdf]

Table S2. GO enrichment of the top 40 hub proteins in Netgsa estimated network.

	GO.ID	Term	Annotated	Significant	Expected	P value
1	GO:0002020	protease binding	35	6	1.19	0.00083
2	GO:0042802	identical protein binding	251	17	8.55	0.00179
3	GO:0004866	endopeptidase inhibitor activity	56	6	1.91	0.00977
4	GO:0005539	glycosaminoglycan binding	93	8	3.17	0.01059
5	GO:0019838	growth factor binding	57	6	1.94	0.01064
6	GO:0030414	peptidase inhibitor activity	57	6	1.94	0.01064
7	GO:0005102	signaling receptor binding	427	22	14.55	0.01117
8	GO:0061135	endopeptidase regulator activity	58	6	1.98	0.01157
9	GO:0031625	ubiquitin protein ligase binding	42	5	1.43	0.01194
10	GO:0004857	enzyme inhibitor activity	77	7	2.62	0.01272
11	GO:0019899	enzyme binding	251	15	8.55	0.01317
12	GO:0061134	peptidase regulator activity	63	6	2.15	0.01711
13	GO:0044389	ubiquitin-like protein ligase binding	46	5	1.57	0.0174
14	GO:0005201	extracellular matrix structural constitu...	33	4	1.12	0.02315
15	GO:0008201	heparin binding	69	6	2.35	0.02592
16	GO:0019904	protein domain specific binding	73	6	2.49	0.03325
17	GO:1901681	sulfur compound binding	80	6	2.73	0.04914

Note:

Annoted, the number of proteins in a pathway within 1212 proteins;

Significant, the number of protens in a pathway within 40 hub proteins;

Expected, the expected number of proteins in a pathway if we randomly selected 40 proteins from 1212 background proteins;

P value: Fisher's test
