# Supplementary material for: An Augmented High-Dimensional Graphical Lasso Method to Incorporate Prior Biological Knowledge for Global Network Learning
Source: Front Genet. 2022 Jan 27;12:760299. doi: 10.3389/fgene.2021.760299 (PMC8829118; doi:10.3389/fgene.2021.760299)
Supplement: Supplementary file 2 [file DataSheet2.ZIP › Frontiers_LaTex_AhGlasso/noPPI_topGo_40_bh.pdf]

Table S1. GO enrichment of the top 40 hub proteins in estimated network without prior PPI knowledge.

	GO.ID	Term	Annotated	Significant	Expected	P value	Adjusted P value
1	GO:0008047	enzyme activator activity	48	7	1.47	0.00041	**0.0455**
2	GO:0019899	enzyme binding	251	15	7.7	0.00421	0.182
3	GO:0003676	nucleic acid binding	136	10	4.17	0.0056	0.182
4	GO:0070851	growth factor receptor binding	76	7	2.33	0.00656	0.182
5	GO:0045296	cadherin binding	47	5	1.44	0.01227	0.2445
6	GO:0030234	enzyme regulator activity	132	9	4.05	0.01449	0.2445
7	GO:0003723	RNA binding	89	7	2.73	0.01542	0.2445
8	GO:0008009	chemokine activity	36	4	1.1	0.02178	0.3022
9	GO:0042379	chemokine receptor binding	39	4	1.2	0.02848	0.3513
10	GO:0031625	ubiquitin protein ligase binding	42	4	1.29	0.0363	0.3587
11	GO:0030546	signaling receptor activator activity	213	11	6.53	0.04677	0.3587
12	GO:0044389	ubiquitin-like protein ligase binding	46	4	1.41	0.0485	0.3587

Note:

Annotated, number of proteins in a pathway from the complete set of 1212 proteins;

Significant, number of protens in a pathway from 40 hub proteins;

Expected, the expected number of proteins in a pathway if we randomly selected 40 proteins from 1212 background proteins;

P value: Fisher'sexact test

Adjusted P value: Benjamini-Hochberg adjusted P value to control for False Discover Rate
