# Supplementary material for: An Augmented High-Dimensional Graphical Lasso Method to Incorporate Prior Biological Knowledge for Global Network Learning
Source: Front Genet. 2022 Jan 27;12:760299. doi: 10.3389/fgene.2021.760299 (PMC8829118; doi:10.3389/fgene.2021.760299)
Supplement: Supplementary file 2 [file DataSheet2.ZIP › Frontiers_LaTex_AhGlasso/netgsa_topGo_40_BH.pdf]

Table S3. GO enrichment of the top 40 hub proteins in Netgsa estimated network.

	GO.ID	Term	Annotated	Significant	Expected	P value	adjusted P value
1	GO:0002020	protease binding	35	6	1.19	0.00083	0.0921
2	GO:0042802	identical protein binding	251	17	8.55	0.00179	0.0993
3	GO:0004866	endopeptidase inhibitor activity	56	6	1.91	0.00977	0.1329
4	GO:0005539	glycosaminoglycan binding	93	8	3.17	0.01059	0.1329
5	GO:0019838	growth factor binding	57	6	1.94	0.01064	0.1329
6	GO:0030414	peptidase inhibitor activity	57	6	1.94	0.01064	0.1329
7	GO:0005102	signaling receptor binding	427	22	14.55	0.01117	0.1329
8	GO:0061135	endopeptidase regulator activity	58	6	1.98	0.01157	0.1329
9	GO:0031625	ubiquitin protein ligase binding	42	5	1.43	0.01194	0.1329
10	GO:0004857	enzyme inhibitor activity	77	7	2.62	0.01272	0.1329
11	GO:0019899	enzyme binding	251	15	8.55	0.01317	0.1329
12	GO:0061134	peptidase regulator activity	63	6	2.15	0.01711	0.1486
13	GO:0044389	ubiquitin-like protein ligase binding	46	5	1.57	0.0174	0.1486
14	GO:0005201	extracellular matrix structural constitu...	33	4	1.12	0.02315	0.1835
15	GO:0008201	heparin binding	69	6	2.35	0.02592	0.1918
16	GO:0019904	protein domain specific binding	73	6	2.49	0.03325	0.2307
17	GO:1901681	sulfur compound binding	80	6	2.73	0.04914	0.3209

Note:

Annotated, number of proteins in a pathway from the complete set of 1212 proteins;

Significant, number of protens in a pathway from 40 hub proteins;

Expected, the expected number of proteins in a pathway if we randomly selected 40 proteins from 1212 background proteins;

P value: Fisher's exact test

Adjusted P value: Benjamini-Hochberg adjusted P value to control for False Discovery Rate
