# Supplementary material for: An Augmented High-Dimensional Graphical Lasso Method to Incorporate Prior Biological Knowledge for Global Network Learning
Source: Front Genet. 2022 Jan 27;12:760299. doi: 10.3389/fgene.2021.760299 (PMC8829118; doi:10.3389/fgene.2021.760299)
Supplement: Supplementary file 2 [file DataSheet2.ZIP › Frontiers_LaTex_AhGlasso/Appendices_COPDGene_Investigators_MAR2021_P2.pdf]

Mortality Adjudication Core: Surya P. Bhatt, MD; Jessica Bon, MD; Alejandro A. Diaz, MD, MPH; MeiLan K. Han, MD, MS; Barry Make, MD; Susan Murray, ScD; Elizabeth Regan, MD; Xavier Soler, MD; Carla G. Wilson, MS

Biomarker Core: Russell P. Bowler, MD, PhD; Katerina Kechris, PhD; Farnoush Banaei-Kashani, PhD

COPDGene® Investigators – Clinical Centers

Ann Arbor VA: Jeffrey L. Curtis, MD; Perry G. Pernicano, MD

Baylor College of Medicine, Houston, TX: Nicola Hanania, MD, MS; Mustafa Atik, MD; Aladin Boriek, PhD; Kalpatha Guntupalli, MD; Elizabeth Guy, MD; Amit Parulekar, MD;

Brigham and Women's Hospital, Boston, MA: Dawn L. DeMeo, MD, MPH; Craig Hersh, MD, MPH; Francine L. Jacobson, MD, MPH; George Washko, MD

Columbia University, New York, NY: R. Graham Barr, MD, DrPH; John Austin, MD; Belinda D'Souza, MD; Byron Thomashow, MD

Duke University Medical Center, Durham, NC: Neil MacIntyre, Jr., MD; H. Page McAdams, MD; Lacey Washington, MD

HealthPartners Research Institute, Minneapolis, MN: Charlene McEvoy, MD, MPH; Joseph Tashjian, MD

Johns Hopkins University, Baltimore, MD: Robert Wise, MD; Robert Brown, MD; Nadia N. Hansel, MD, MPH; Karen Horton, MD; Allison Lambert, MD, MHS; Nirupama Putcha, MD, MHS

Lundquist Institute for Biomedical Innovation at Harbor UCLA Medical Center, Torrance, CA: Richard Casaburi, PhD, MD; Alessandra Adami, PhD; Matthew Budoff, MD; Hans Fischer, MD; Janos Porszasz, MD, PhD; Harry Rossiter, PhD; William Stringer, MD

Michael E. DeBakey VAMC, Houston, *TX*: Amir Sharafkhaneh, MD, PhD; Charlie Lan, DO

Minneapolis VA: Christine Wendt, MD; Brian Bell, MD; Ken M. Kunisaki, MD, MS

Morehouse School of Medicine, Atlanta, GA: Eric L. Flenaugh, MD; Hirut Gebrekristos, PhD; Mario Ponce, MD; Silanath Terpenning, MD; Gloria Westney, MD, MS

National Jewish Health, Denver, CO: Russell Bowler, MD, PhD; David A. Lynch, MB

Reliant Medical Group, Worcester, MA: Richard Rosiello, MD; David Pace, MD

Temple University, Philadelphia, PA: Gerard Criner, MD; David Ciccolella, MD; Francis Cordova, MD; Chandra Dass, MD; Gilbert D'Alonzo, DO; Parag Desai, MD; Michael Jacobs, PharmD; Steven Kelsen, MD, PhD; Victor Kim, MD; A. James Mamary, MD; Nathaniel

Marchetti, DO; Aditi Satti, MD; Kartik Shenoy, MD; Robert M. Steiner, MD; Alex Swift, MD; Irene Swift, MD; Maria Elena Vega-Sanchez, MD

University of Alabama, Birmingham, AL: Mark Dransfield, MD; William Bailey, MD; Surya P. Bhatt, MD; Anand Iyer, MD; Hrudaya Nath, MD; J. Michael Wells, MD

University of California, San Diego, CA: Douglas Conrad, MD; Xavier Soler, MD, PhD; Andrew Yen, MD

University of Iowa, Iowa City, IA: Alejandro P. Comellas, MD; Karin F. Hoth, PhD; John Newell, Jr., MD; Brad Thompson, MD

University of Michigan, Ann Arbor, MI: MeiLan K. Han, MD MS; Ella Kazerooni, MD MS; Wassim Labaki, MD MS; Craig Galban, PhD; Dharshan Vummidi, MD

University of Minnesota, Minneapolis, MN: Joanne Billings, MD; Abbie Begnaud, MD; Tadashi Allen, MD

University of Pittsburgh, Pittsburgh, PA: Frank Sciurba, MD; Jessica Bon, MD; Divay Chandra, MD, MSc; Joel Weissfeld, MD, MPH

University of Texas Health, San Antonio, San Antonio, TX: Antonio Anzueto, MD; Sandra Adams, MD; Diego Maselli-Caceres, MD; Mario E. Ruiz, MD; Harjinder Singh
