# Supplementary material for: An Augmented High-Dimensional Graphical Lasso Method to Incorporate Prior Biological Knowledge for Global Network Learning
Source: Front Genet. 2022 Jan 27;12:760299. doi: 10.3389/fgene.2021.760299 (PMC8829118; doi:10.3389/fgene.2021.760299)
Supplement: Supplementary file 2 [file DataSheet2.ZIP › Frontiers_LaTex_AhGlasso/figures_thesis/Data_simulation.pdf]

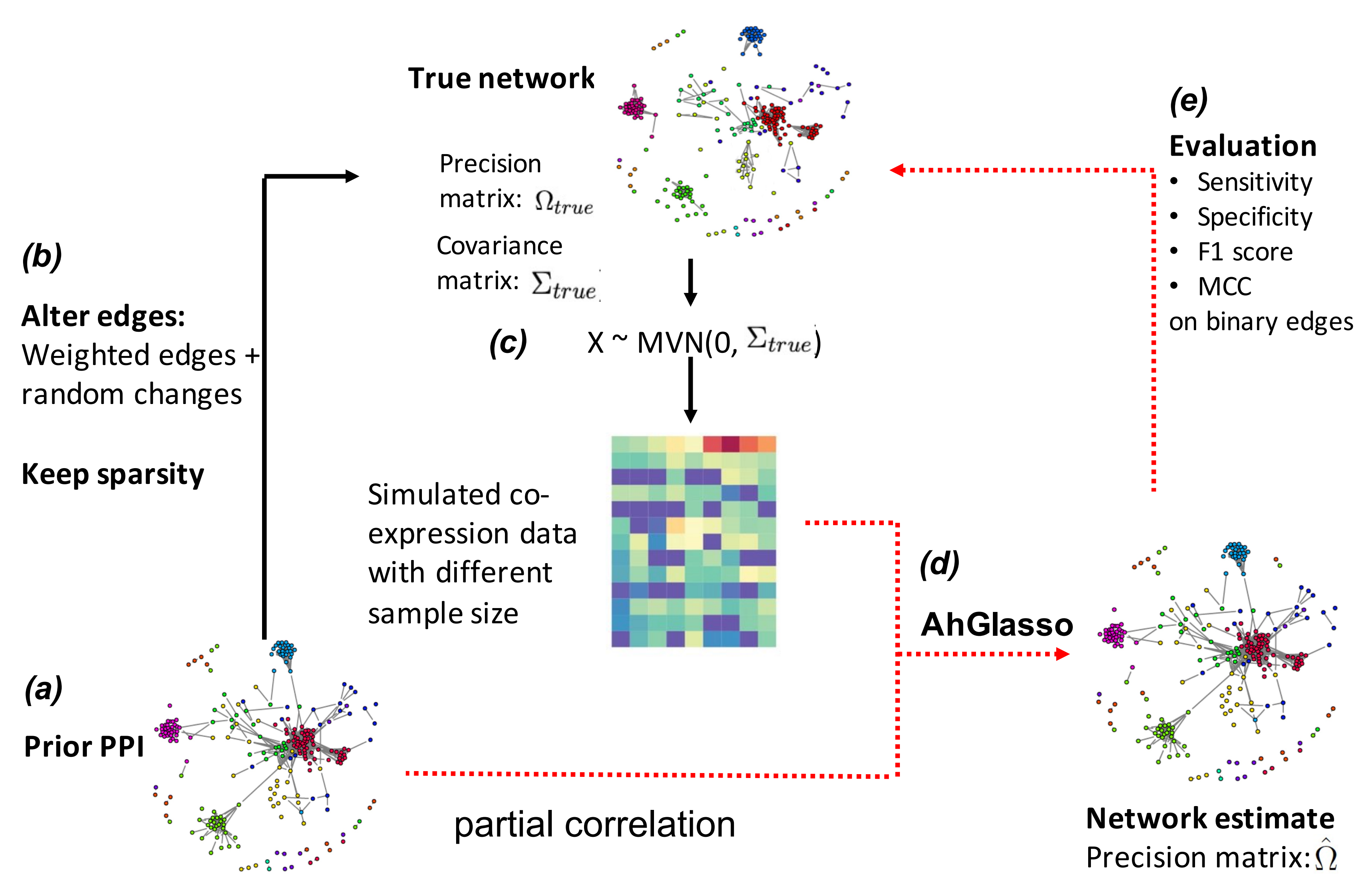
True network
Precision matrix: Ω_{true}
Covariance matrix: Σ_{true}
(b)
Alter edges:
Weighted edges + random changes
Keep sparsity
(c)
X ~ MVN(0, Σ_{true})
Simulated co-expression data with different sample size
(e)
Evaluation
• Sensitivity
• Specificity
• F1 score
• MCC
on binary edges
(d)
AhGlasso
(a)
Prior PPI
partial correlation
Network estimate
Precision matrix: $\hat{\Omega}$
