# Supplementary material for: An Augmented High-Dimensional Graphical Lasso Method to Incorporate Prior Biological Knowledge for Global Network Learning
Source: Front Genet. 2022 Jan 27;12:760299. doi: 10.3389/fgene.2021.760299 (PMC8829118; doi:10.3389/fgene.2021.760299)
Supplement: Supplementary file 2 [file DataSheet2.ZIP › Frontiers_LaTex_AhGlasso/figures_thesis/netgsa_topGo_40.pdf]

**Table S2. GO enrichment of the top 40 hub proteins in Netgsa estimated network.**

| | GO.ID | Term | Annotated | Significant | Expected | P value |
|---|---|---|---|---|---|---|
| 1 | GO:0002020 | protease binding | 35 | 6 | 1.19 | 0.00083 |
| 2 | GO:0042802 | identical protein binding | 251 | 17 | 8.55 | 0.00179 |
| 3 | GO:0004866 | endopeptidase inhibitor activity | 56 | 6 | 1.91 | 0.00977 |
| 4 | GO:0005539 | glycosaminoglycan binding | 93 | 8 | 3.17 | 0.01059 |
| 5 | GO:0019838 | growth factor binding | 57 | 6 | 1.94 | 0.01064 |
| 6 | GO:0030414 | peptidase inhibitor activity | 57 | 6 | 1.94 | 0.01064 |
| 7 | GO:0005102 | signaling receptor binding | 427 | 22 | 14.55 | 0.01117 |
| 8 | GO:0061135 | endopeptidase regulator activity | 58 | 6 | 1.98 | 0.01157 |
| 9 | GO:0031625 | ubiquitin protein ligase binding | 42 | 5 | 1.43 | 0.01194 |
| 10 | GO:0004857 | enzyme inhibitor activity | 77 | 7 | 2.62 | 0.01272 |
| 11 | GO:0019899 | enzyme binding | 251 | 15 | 8.55 | 0.01317 |
| 12 | GO:0061134 | peptidase regulator activity | 63 | 6 | 2.15 | 0.01711 |
| 13 | GO:0044389 | ubiquitin-like protein ligase binding | 46 | 5 | 1.57 | 0.0174 |
| 14 | GO:0005201 | extracellular matrix structural constitu... | 33 | 4 | 1.12 | 0.02315 |
| 15 | GO:0008201 | heparin binding | 69 | 6 | 2.35 | 0.02592 |
| 16 | GO:0019904 | protein domain specific binding | 73 | 6 | 2.49 | 0.03325 |
| 17 | GO:1901681 | sulfur compound binding | 80 | 6 | 2.73 | 0.04914 |

**Note:**

Annotated, number of proteins in a pathway from the complete set of 1212 proteins;

Significant, number of protens in a pathway from 40 hub proteins;

Expected, the expected number of proteins in a pathway if we randomly selected 40 proteins from 1212 background proteins;

P value: Fisher'sexact test
