# Supplementary figures and images for: An Augmented High-Dimensional Graphical Lasso Method to Incorporate Prior Biological Knowledge for Global Network Learning
Source: Front Genet. 2022 Jan 27;12:760299. doi: 10.3389/fgene.2021.760299 (PMC8829118; doi:10.3389/fgene.2021.760299)

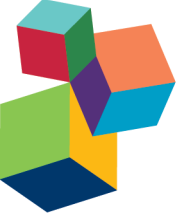

frontiers

Supplement: Supplementary file 2 [file DataSheet2.ZIP › Frontiers_LaTex_AhGlasso/logo1-eps-converted-to.pdf]

A

frontiers  
FOR YOUNG MINDS

B

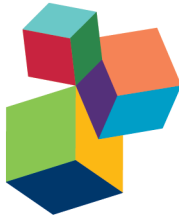

Supplement: Supplementary file 2 [file DataSheet2.ZIP › Frontiers_LaTex_AhGlasso/logos-eps-converted-to.pdf]

**A**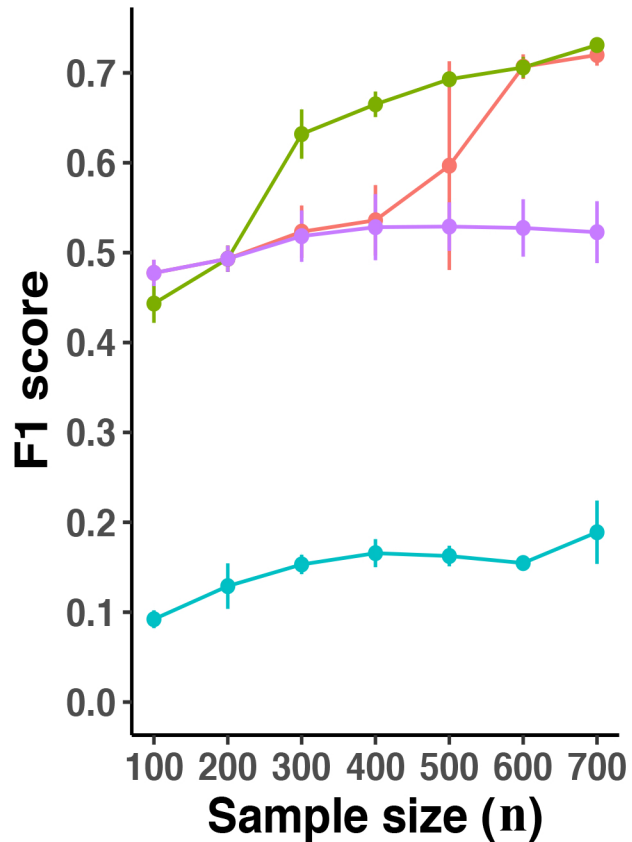**B**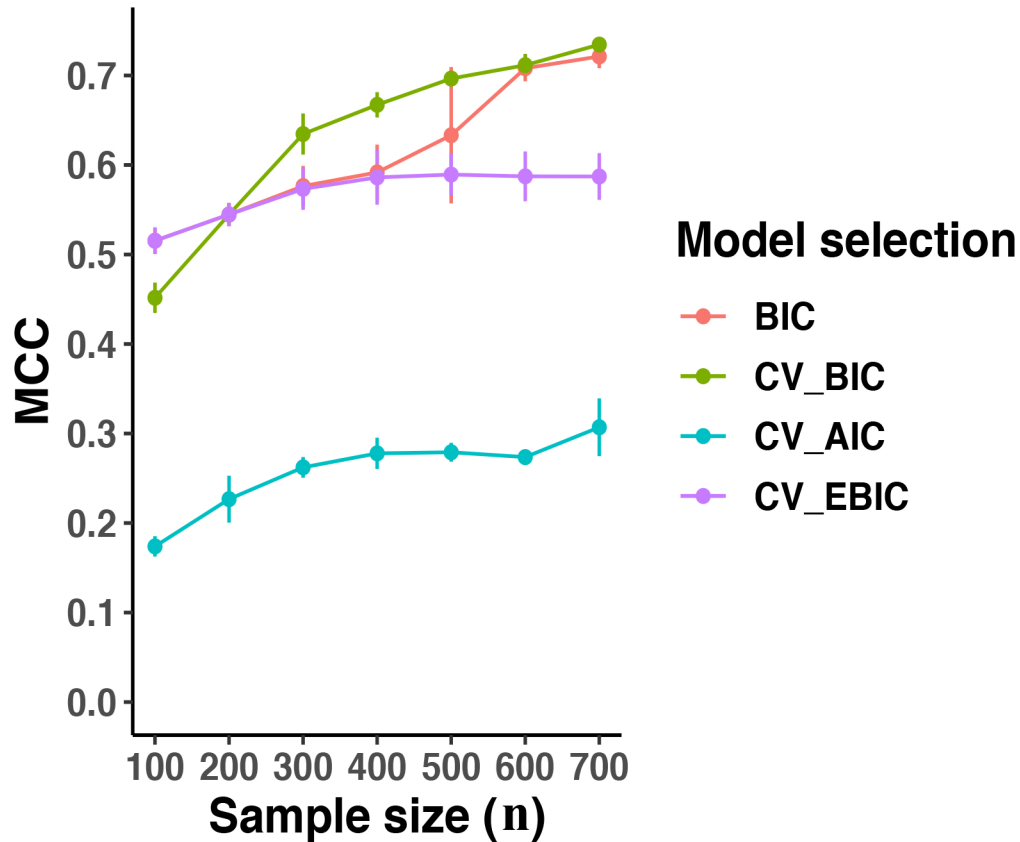

Supplement: Supplementary file 2 [file DataSheet2.ZIP › Frontiers_LaTex_AhGlasso/figures_thesis/modelSelection_1.pdf]

**A**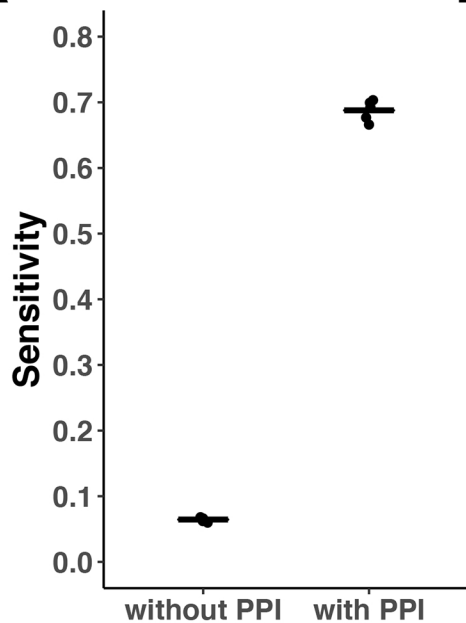**B**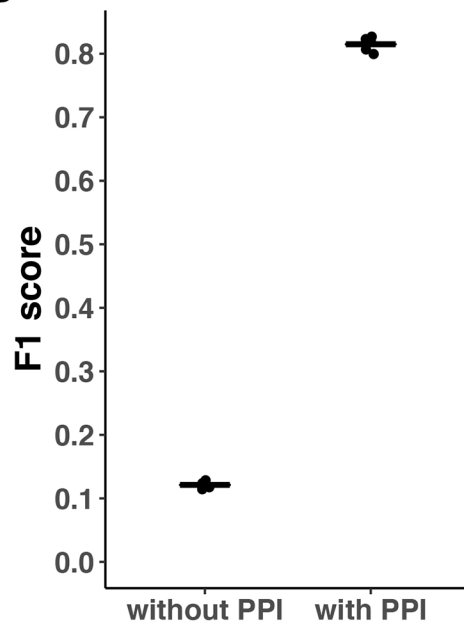**C**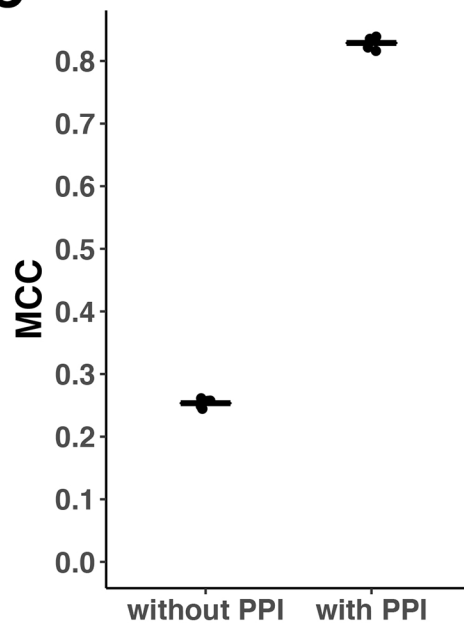**D**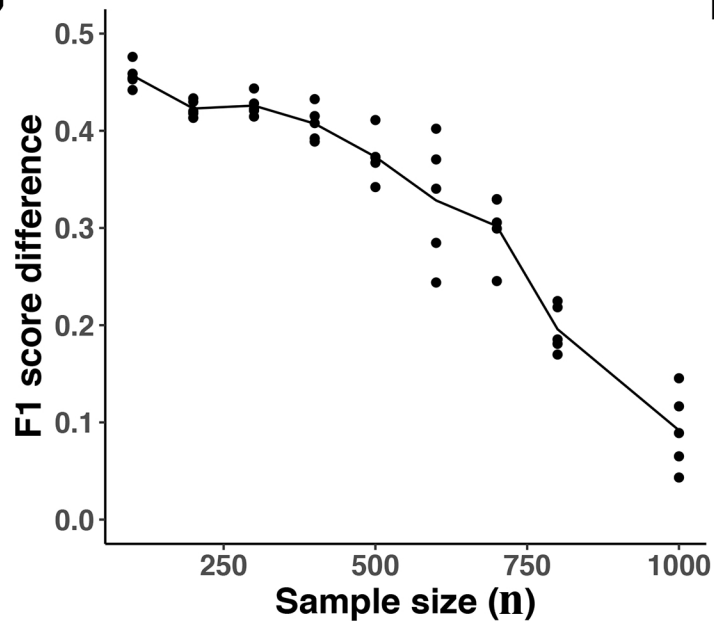**E**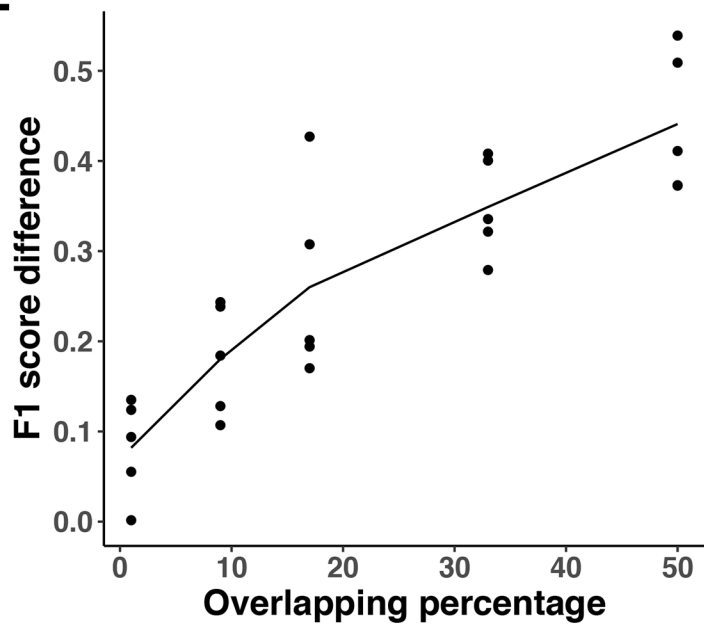

Supplement: Supplementary file 2 [file DataSheet2.ZIP › Frontiers_LaTex_AhGlasso/figures_thesis/ppiAdvantages.pdf]

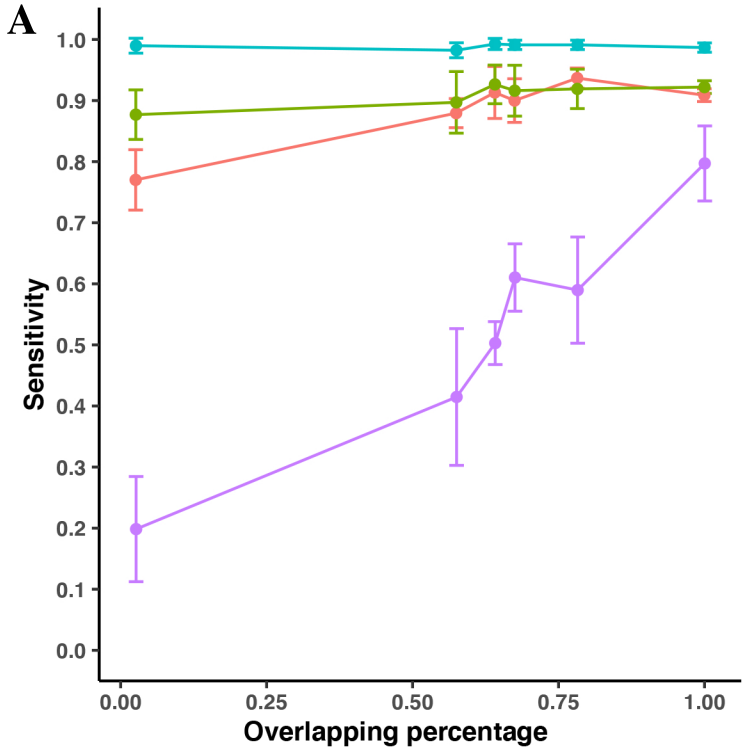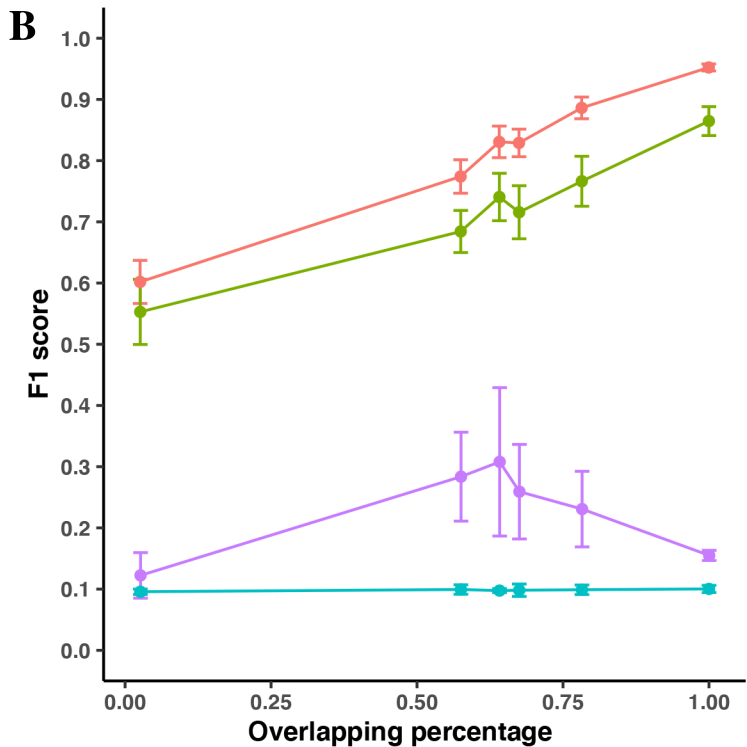

Supplement: Supplementary file 2 [file DataSheet2.ZIP › Frontiers_LaTex_AhGlasso/figures_thesis/MethodComparison_F1_overlapping_random.pdf]

**A**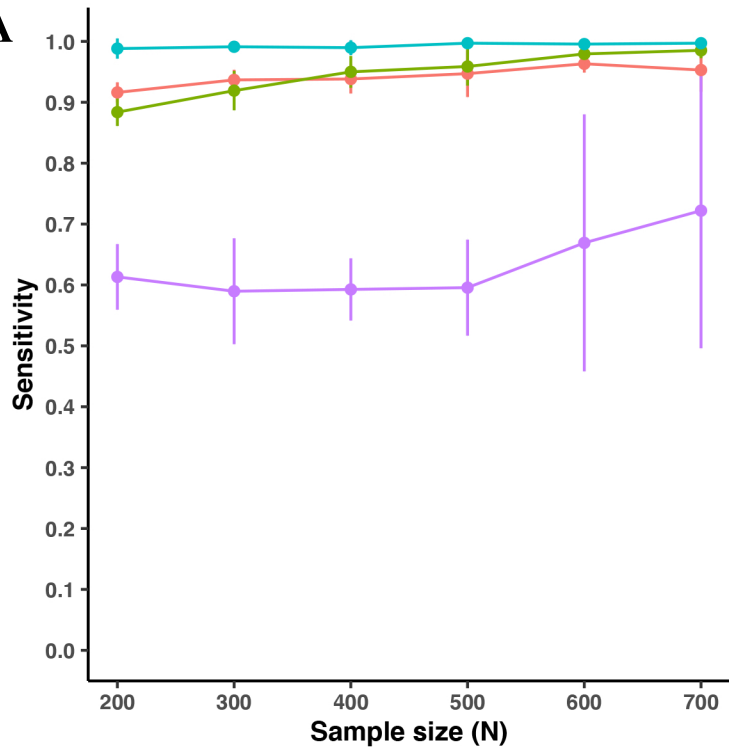**B**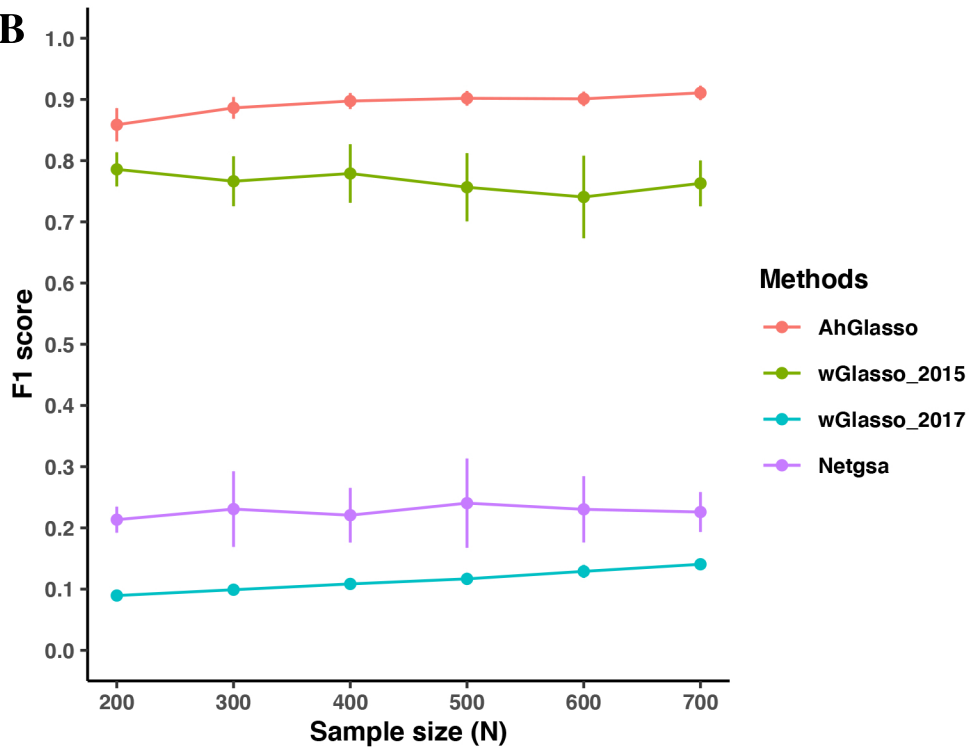

Supplement: Supplementary file 2 [file DataSheet2.ZIP › Frontiers_LaTex_AhGlasso/figures_thesis/MethodComparison_F1_random.pdf]

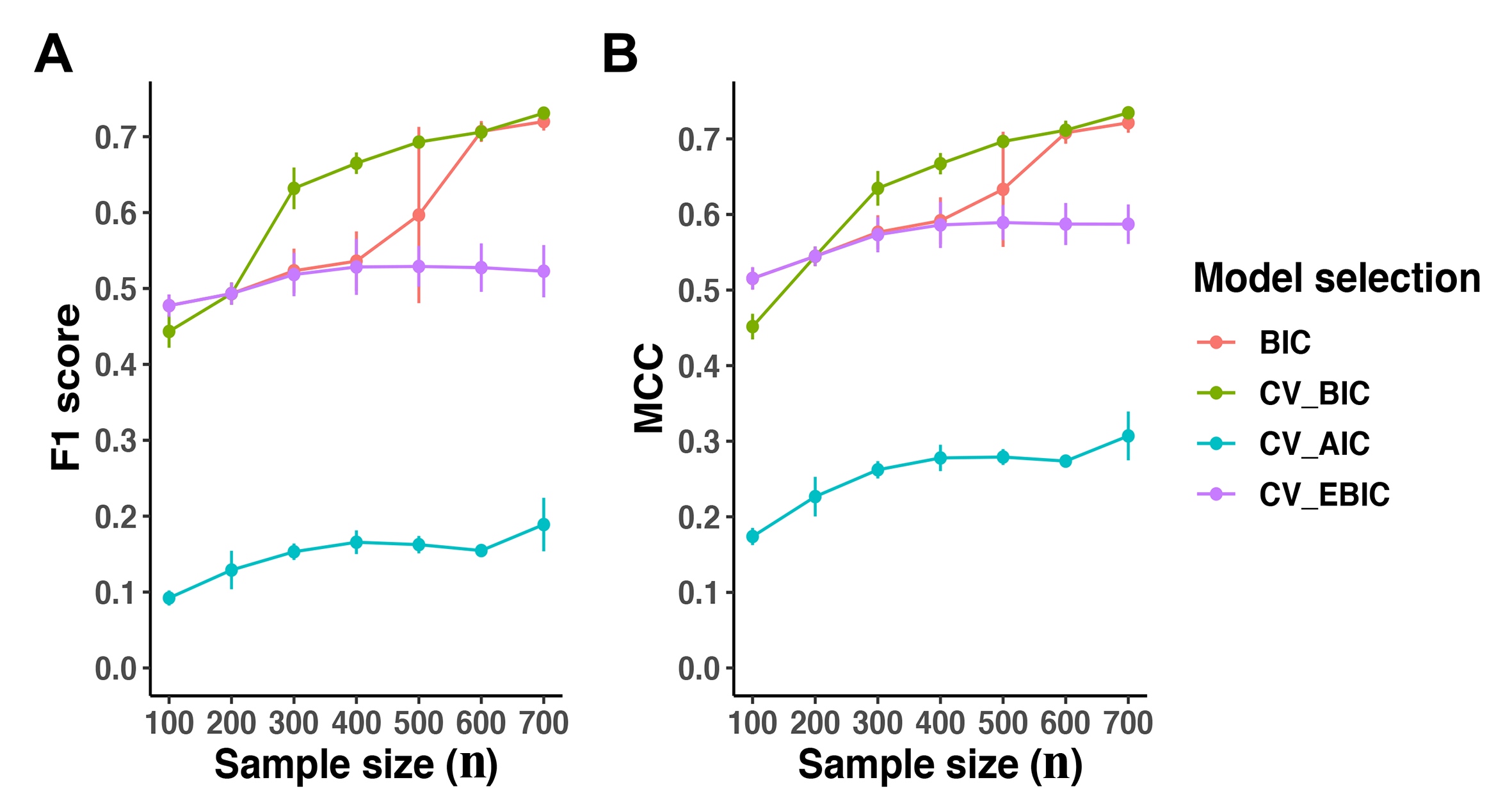

Supplement: Supplementary file 2 [file DataSheet2.ZIP › Frontiers_LaTex_AhGlasso/figures_thesis/Fig2_modelSelection_1.tif]

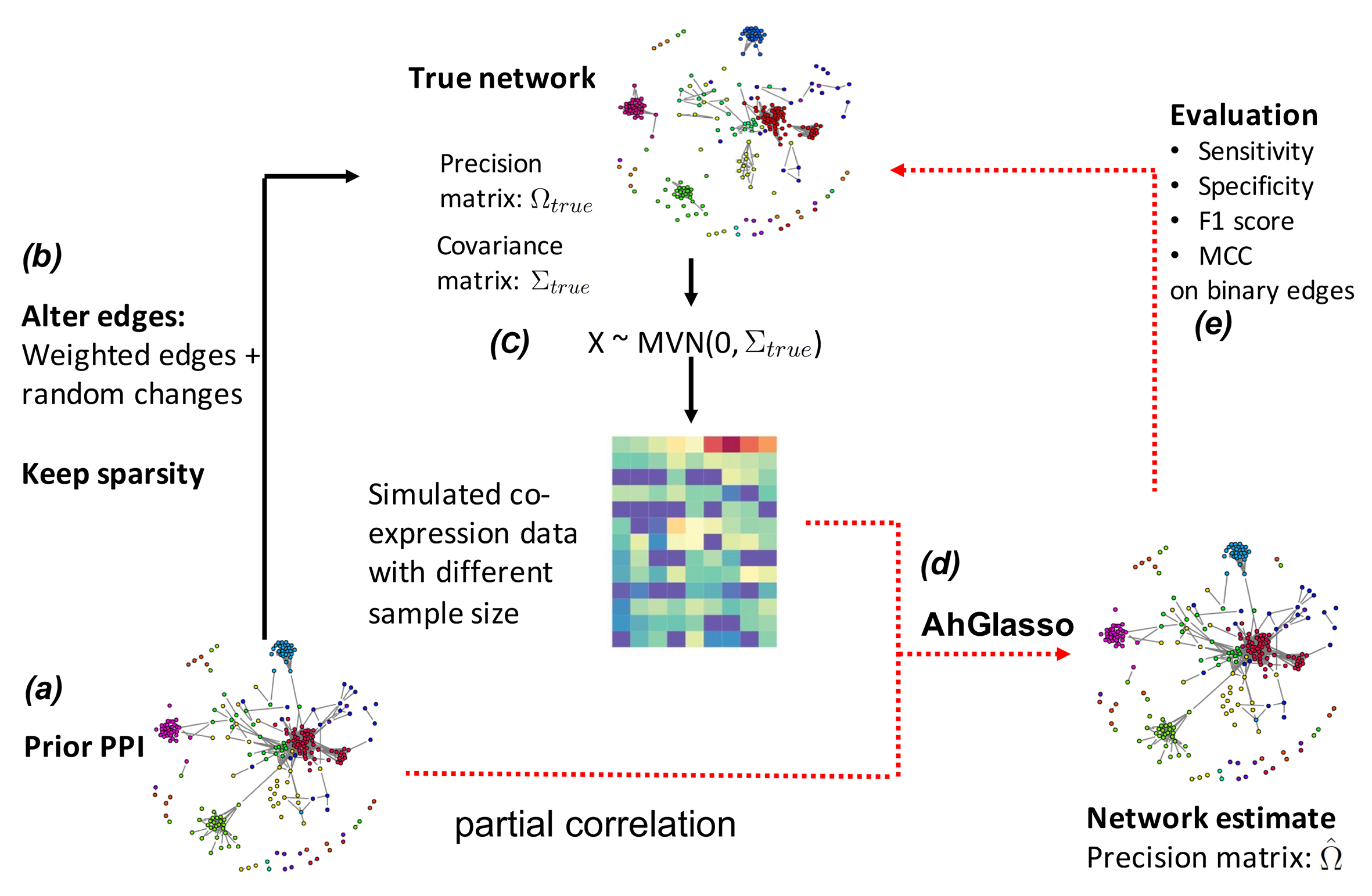

Supplement: Supplementary file 2 [file DataSheet2.ZIP › Frontiers_LaTex_AhGlasso/figures_thesis/Fig1_Data_simulation.tif]

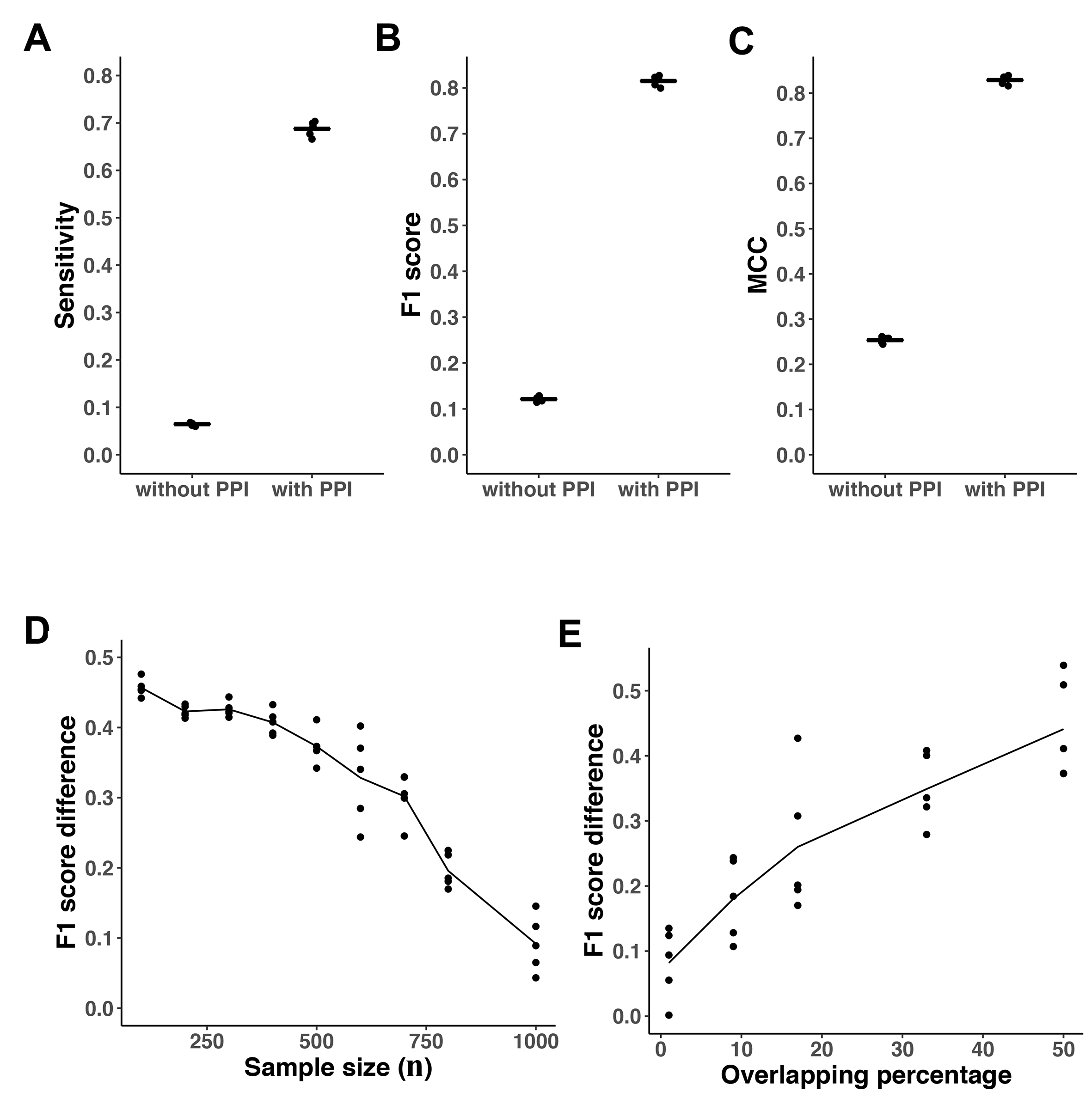

Supplement: Supplementary file 2 [file DataSheet2.ZIP › Frontiers_LaTex_AhGlasso/figures_thesis/Fig3_ppiAdvantages.tif]

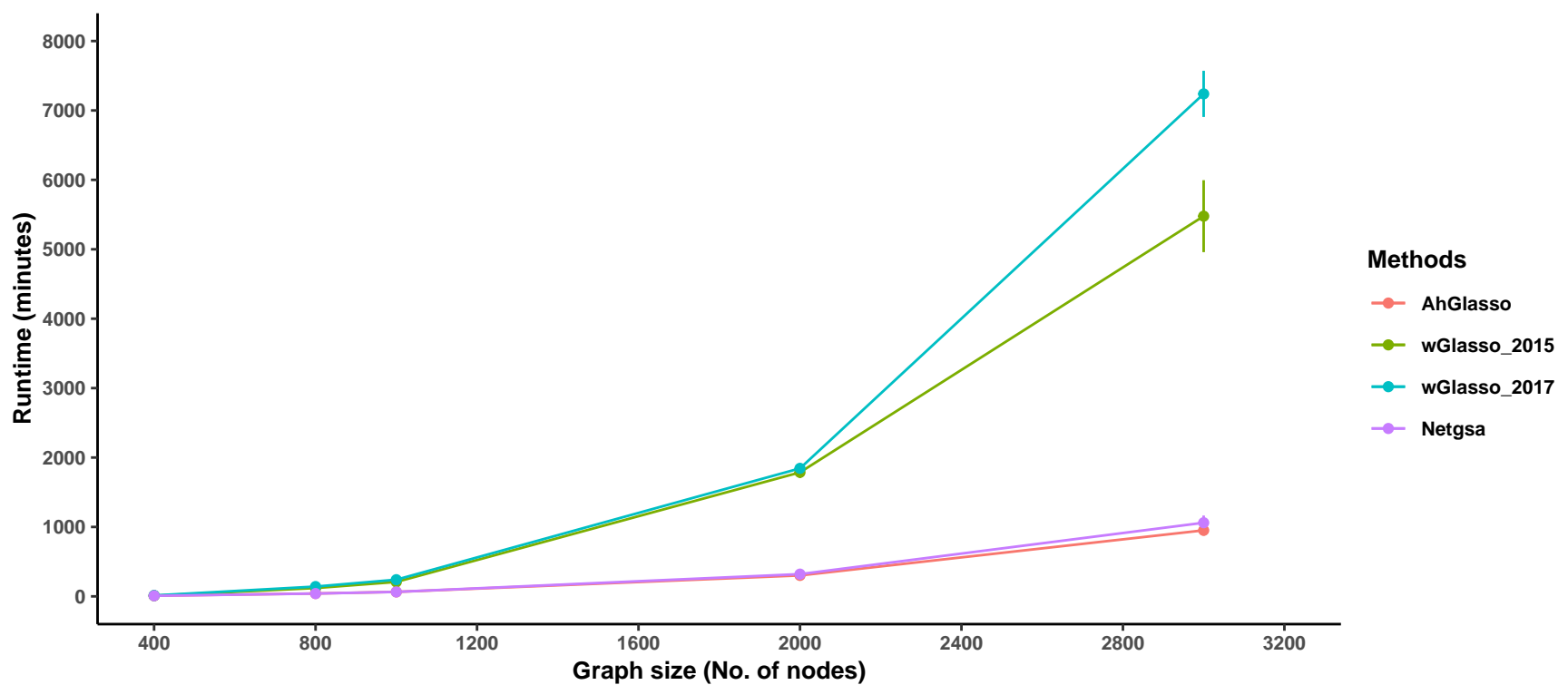

Supplement: Supplementary file 2 [file DataSheet2.ZIP › Frontiers_LaTex_AhGlasso/figures_thesis/MethodComparison_runtime_differentGraph_size.pdf]

**A**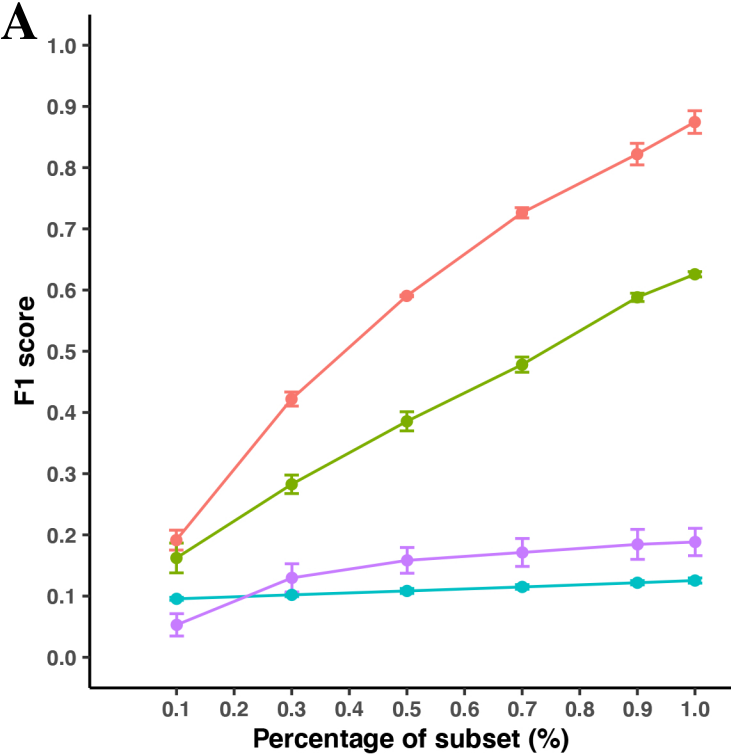**B**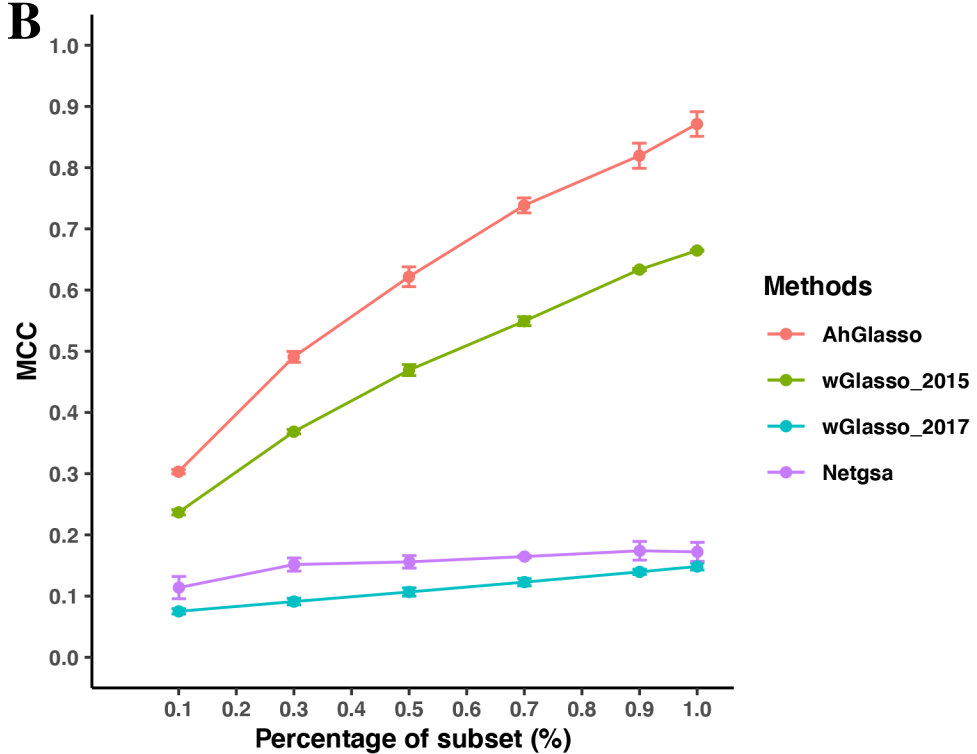

Supplement: Supplementary file 2 [file DataSheet2.ZIP › Frontiers_LaTex_AhGlasso/figures_thesis/MethodComparison_F1_subset.pdf]

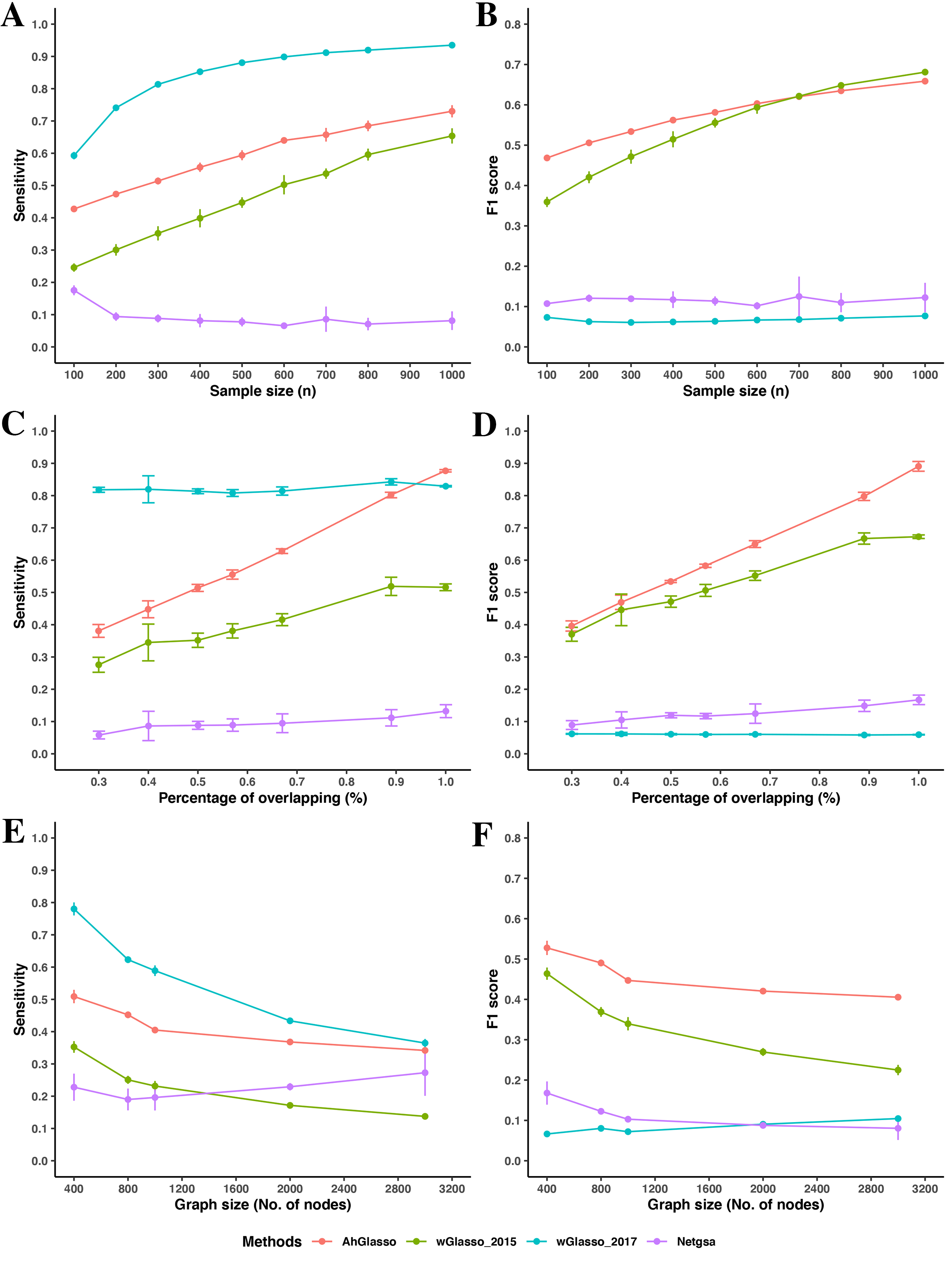

Supplement: Supplementary file 2 [file DataSheet2.ZIP › Frontiers_LaTex_AhGlasso/figures_thesis/Fig4_MethodComparison_F1_diffOverlappingSamplesize_6f_rev.tif]

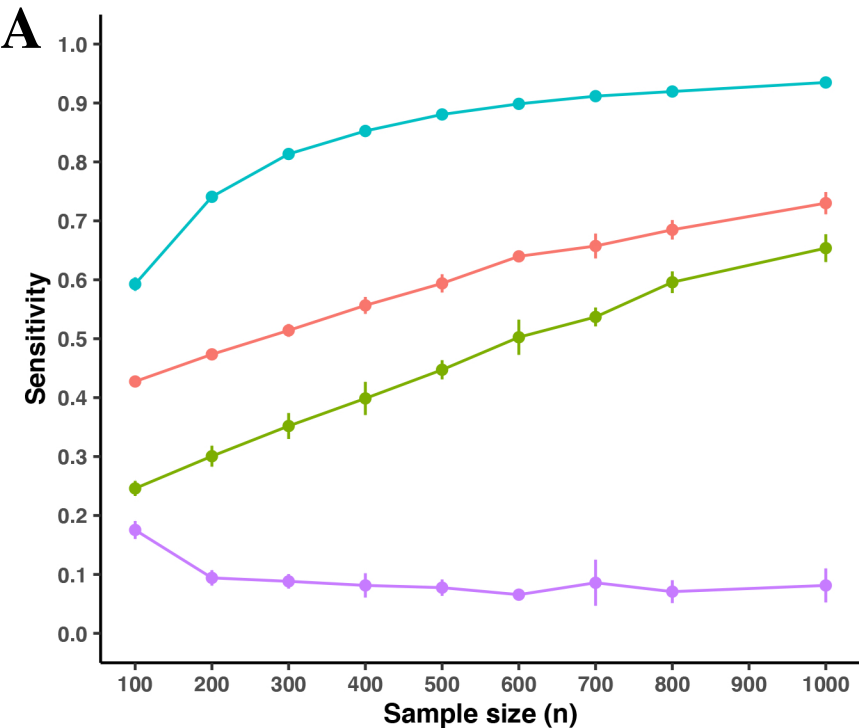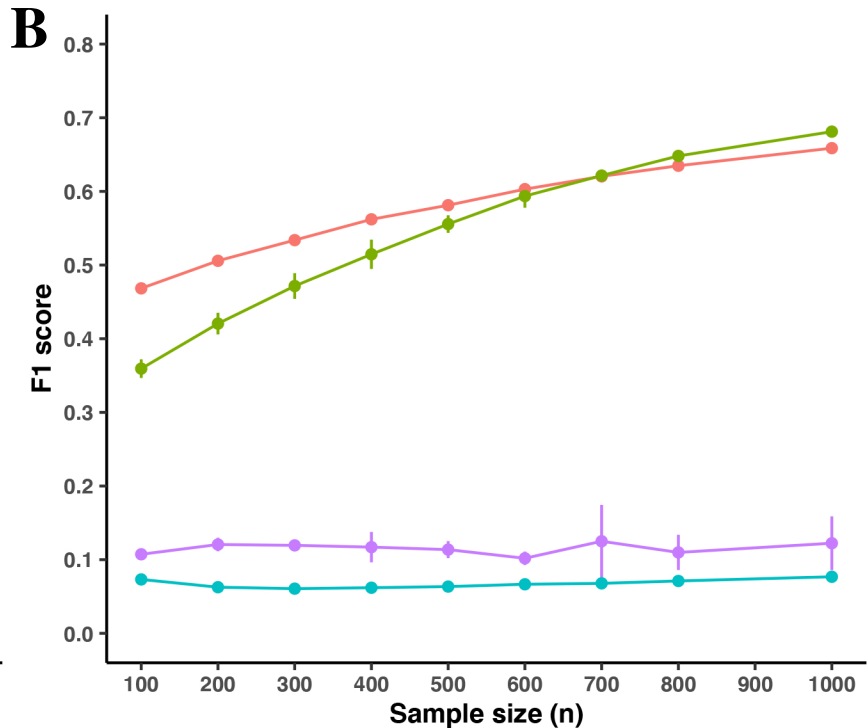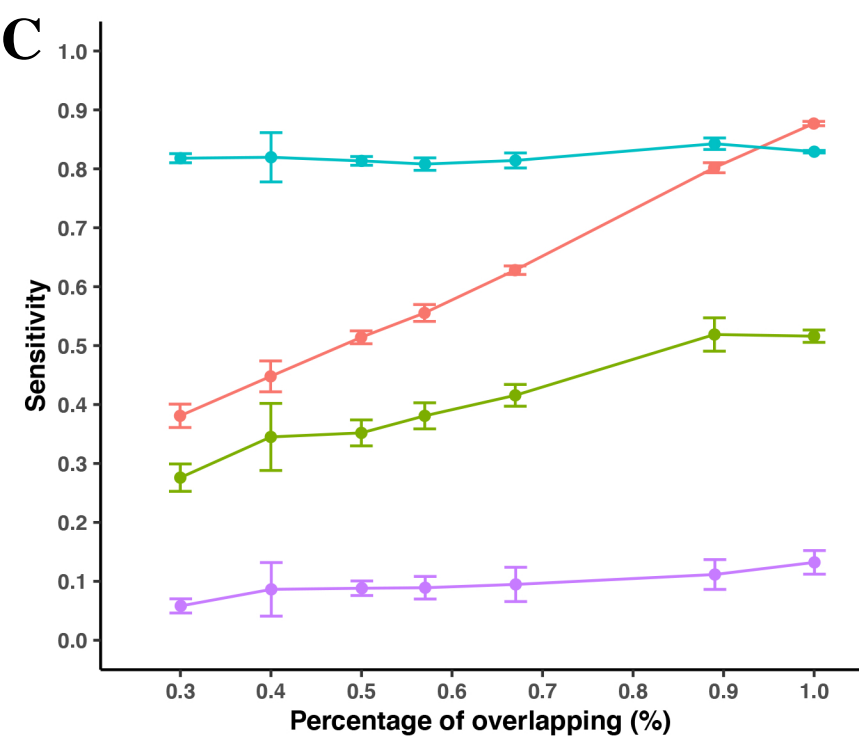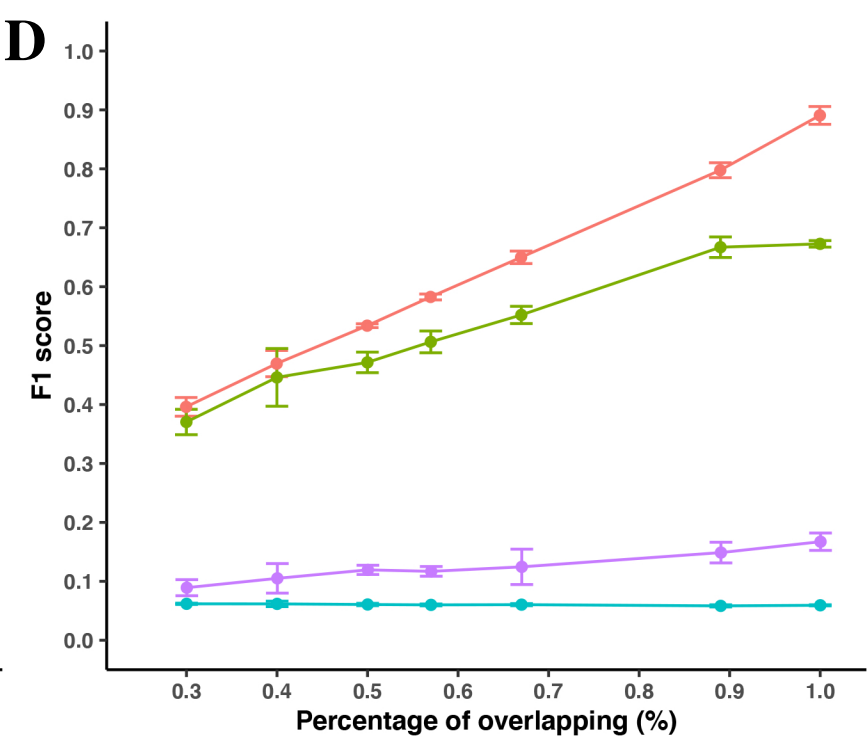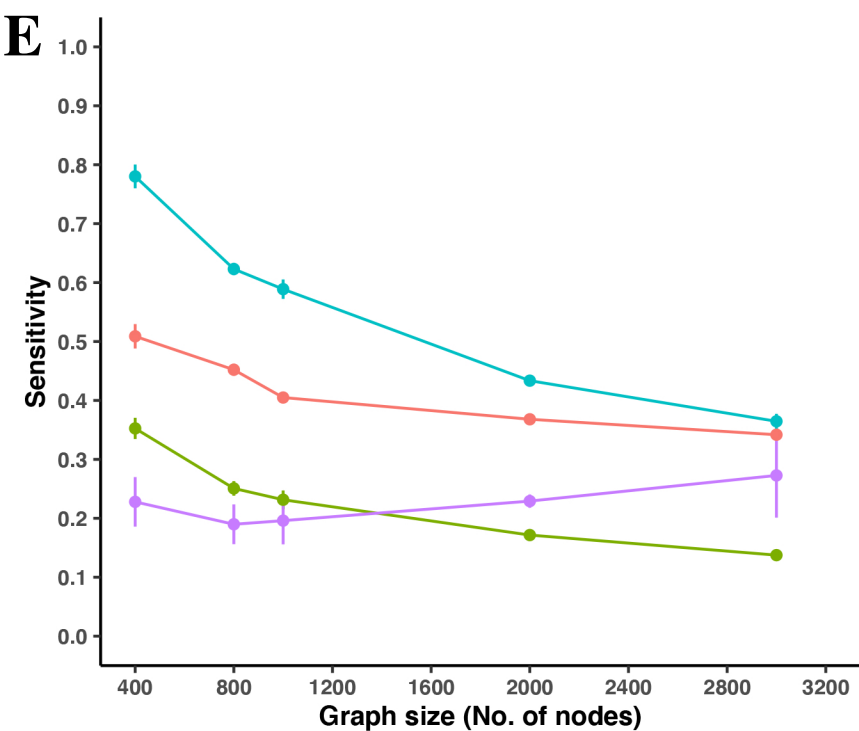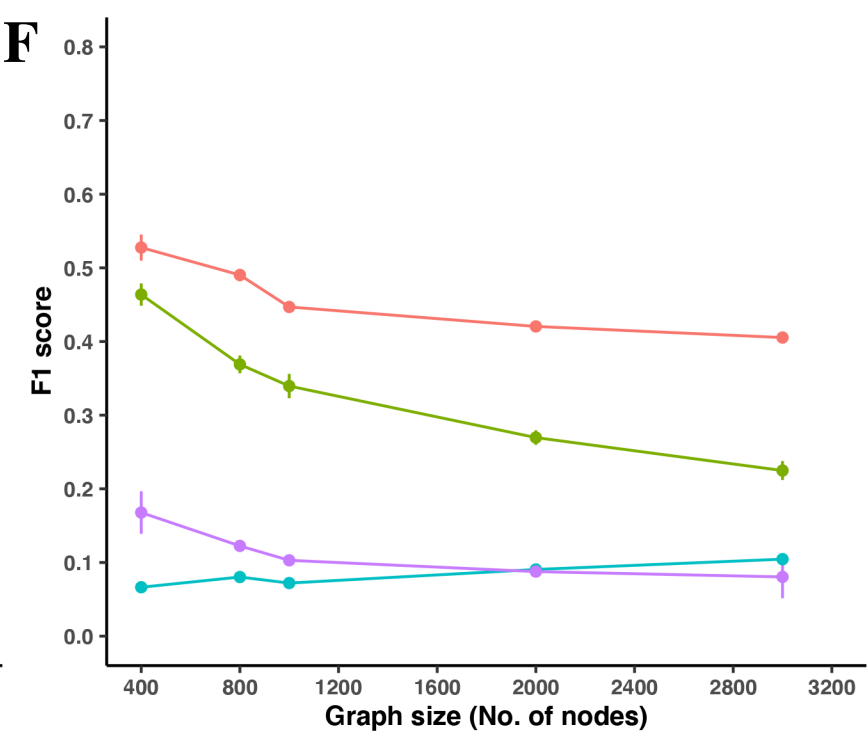

Methods — AhGlasso — wGlasso\_2015 — wGlasso\_2017 — Netgsa

Supplement: Supplementary file 2 [file DataSheet2.ZIP › Frontiers_LaTex_AhGlasso/figures_thesis/MethodComparison_F1_diffOverlappingSamplesize_6f_rev.pdf]

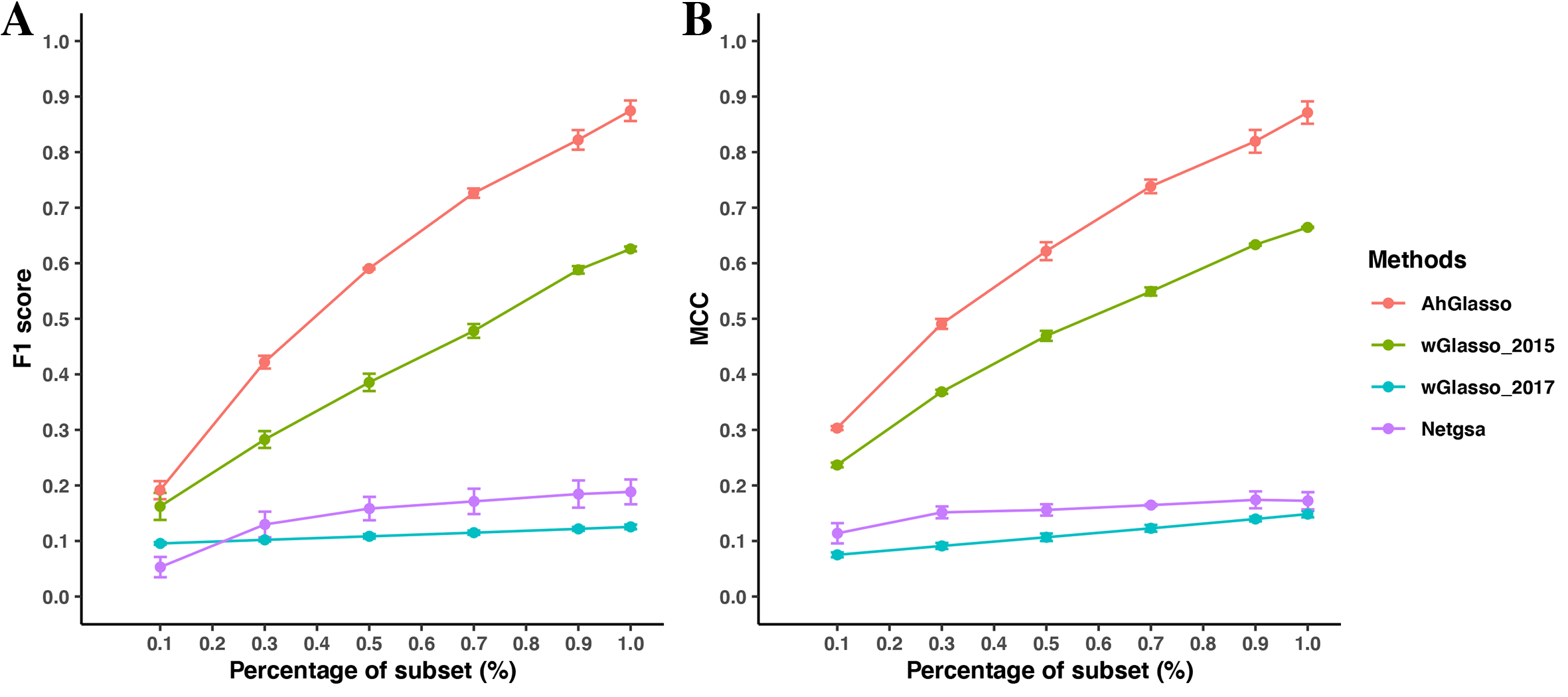

Supplement: Supplementary file 2 [file DataSheet2.ZIP › Frontiers_LaTex_AhGlasso/figures_thesis/Fig5_MethodComparison_F1_subset.tif]

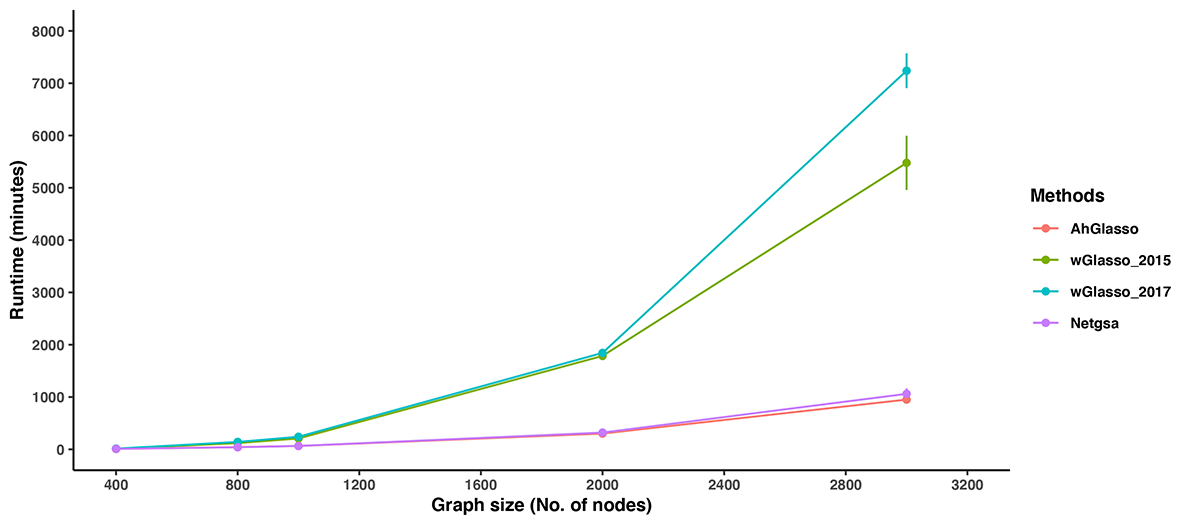

Supplement: Supplementary file 2 [file DataSheet2.ZIP › Frontiers_LaTex_AhGlasso/figures_thesis/Fig6_MethodComparison_runtime_differentGraph_size.tif]
